# Generating three-dimensional genome structures with a variational quantum algorithm

**DOI:** 10.1093/bib/bbaf663

**Published:** 2025-12-15

**Authors:** Andrew Jordan Siciliano, Zheng Wang

**Affiliations:** Department of Computer Science, University of Miami, 1365 Memorial Drive, Coral Gables 33124, FL, United States; Department of Computer Science, University of Miami, 1365 Memorial Drive, Coral Gables 33124, FL, United States

**Keywords:** chromosome conformation capture, chromosome modeling, variational quantum algorithms, quantum computing

## Abstract

Chromosome conformation capture experiments have revealed the underlying spatial interactions that govern three-dimensional (3D) genome organization and topology. Detecting 3D contacts between genomic loci considerably enhances our understanding of fundamental regulatory processes. Modeling 3D structures from experimental contact matrices can further contextualize the relationship between 3D genome organization and regulation. While classical algorithms have been successful in reconstructing genomic conformations, we investigate the prospect of quantum computation to aid in modeling the conformational space. In this context, we propose a novel variational quantum algorithm (VQA) to model the distribution of 3D genomic structures from experimental contact data. Through rigorous evaluations, we demonstrate the capability of our algorithm to sample ensembles of viable 3D conformations that agree well with experimental and simulated contact data. Furthermore, we extend our methodology to model the conformational space of a single cell or a population of cells. In the advent of sufficient quantum utility, the insights gained from this study can serve as a foundation for investigating high-resolution, large-scale ensembles of genomic conformations through generative VQAs.

## Introduction

Chromosome conformation capture (3C) experiments detect topological and organizational properties of the entire physical genome [1], and captured contacts relate directly to the spatial proximity between pairs of genomic loci. More recently developed experimental protocols, such as the Hi-C [2, 3] and Micro-C [4] experiments, have provided the underlying spatial organization of entire genomes with unprecedented details. Single-cell Hi-C [5] is an extension of the Hi-C experiment that provides us with contacts at the level of individual cells. Single-cell genomic conformations have a high degree of variability [5], with respective contacts providing a deeper understanding of cell-cycle dynamics [6] and genome regulation processes [7]. 3C experiments and their derivatives reveal structural features of chromatin organization, such as topological domains [8] and chromatin loops [9]. These features have proven invaluable for understanding promoter–promoter and promoter–enhancer interactions, gene activation processes, gene expression, transcriptional regulation, and disease development and progression [8–27].

Captured genomic contacts are inversely correlated with pairwise 3D target distances. We can use inverse exponential functions, such as $d[i,j] = f[i,j]^{-\frac{1}{3}}$ with $d[i,j]$ being the target distance and $f[i,j]$ being the contact frequency captured between loci $i$ and $j$, to produce target distance matrices (distograms) of the full genomic structures [28–30]. However, to understand the relations between genomic structure and function, modeling (simulating) the structure of 3D genomes is necessary to gain further insights [31]. For example, structural models can help decipher the relationship between 3D conformations and gene regulation, gene expression, and enhancer activities [31–36]. Computational methods have been successful in approximately reconstructing the spatial locations of 3D genomic structures from the captured contacts (or target distances), with common approaches including numerical optimization and simulated annealing [27–30, 36–43].

Many 3D conformations can fit the experimentally determined contacts, thus producing ensembles of viable genomic conformations is of high interest. Ensembles are commonly generated via simultaneous optimization routines, such as in deconvolution methods [35, 41], or repeated runs of a proposed algorithm from different starting points or random seeds, such as in sampling methods [38, 44]. Probabilistic modeling has also been shown to be effective in producing diverse structural ensembles [41, 42, 44]. However, many approaches become computationally expensive due to lengthy simulation (optimization) times or large memory requirements. Oftentimes, generating even moderately sized ensembles can demand significant resource utilization, especially so for high-resolution contact data.

In the disciplines of bioinformatics and computational biology, quantum computing has, in recent times, begun to be explored [45–49]. Many proposed algorithms offer promising utility. For example, quantum algorithms have been proposed for *de novo* genome assembly [50–52], mRNA codon optimization [53], the RNA folding problem [54, 55], and protein binding site identification [56]. Notably, an adaptation of the Variational Quantum Eigensolver [57] that utilizes Conditional Value-at-Risk (CVaR) [58] has been applied to the protein folding problem. The model proposed by Robert *et al.* [59] was able to successfully fold small peptides on a tetrahedral lattice, with their proposed algorithm achieving results comparable to those obtained through X-ray crystallography [60]. However, the feasibility of tackling the protein folding problem on near-term devices is still an open problem with potential barriers being explored [61, 62].

Variational quantum algorithms (VQAs) are heuristic-based algorithms heavily researched for quantum chemistry and combinatorial optimization. VQAs involve both classical and quantum computation, linked in a feedback loop. An objective function $\mathcal{L}$ is computed over a set of sampled measurements, and parameterized circuits (ansatz) are variationally adjusted to optimize $\mathcal{L}$. The applications of VQAs on Noisy Intermediate Scale Quantum [63] devices are suggested to be promising, due to shallow circuit depth requirements and some resilience to noise [64]. However, issues of barren plateaus [65, 66] and objective function landscape difficulties [67, 68] are intrinsic problems associated with training VQAs. It is still unclear whether advantages in the near term are feasible [69]; however, potential mitigation strategies are being explored with some success [70–78]. Even with the aforementioned obstacles, given improvements in hardware and error correction technologies, the prospective real-world usefulness of VQAs could be wide-reaching [79, 80].

The Variational Quantum Eigensolver [57] (VQE) is a fundamental VQA, which attempts to approximate the ground (lowest energy) state of a given Hamiltonian $\mathcal{H}$. The VQE can be used for combinatorial optimization problems when $\mathcal{H}$ is equivalent to a pseudo-boolean polynomial [81]. A parameterized quantum state is prepared using an ansatz circuit, with parameters variationally adjusted to minimize the expectation of the energy of the system. Typically, $\mathcal{H}$ is decomposed into a set of Pauli strings, and the expected energy is computed using the Hamiltonian averaging technique [57, 82]. The procedure for a wide variety of VQAs is similar to that of VQE, with the difference being how the objective function is defined and computed. While the goal of VQE is to approximate the ground state of the Hamiltonian, sometimes it is preferred to model a specific target distribution, such as in quantum generative modeling. We can define an objective function whose minimum guides the quantum state toward the target distribution of interest. Objective functions need not be entirely classical and can also be designed to compare sample data points using kernel and discrepancy methods [83].

Quantum machine learning [84] has applications for both supervised and unsupervised learning. It is natural to employ quantum systems as generative models, as they are fundamentally probabilistic. Quantum generative models (QGMs) are VQAs that aim to embed a target distribution in the quantum system using parameterized circuits. It has been shown that under certain conditions, QGMs can provide clear benefits with respect to expressibility and sampling efficiency [85–87]. Determining the long-term viability of VQAs in the context of generative modeling is still an open problem; however, current research has suggested promising insights and perspectives on the future applications of QGMs [83, 88–90].

We developed a VQA that aims to model the conformational space of 3D genomic structures. To the best of our knowledge, this is the first time quantum computing has been proposed for the 3D genome reconstruction problem. We implemented and investigated a mathematical model specifically designed to simulate high-likelihood ensembles of genomic conformations from bulk or single-cell Hi-C data on a quantum computer. In the advent of sufficient quantum utility [63, 91], our proposed algorithm could offer further insights into the space of viable 3D genomic conformations, advancing our understanding of 3D genomic organization and its role in various biological processes.

## Materials and methods

### Problem statement and objective function

We formulate the 3D genome reconstruction problem in terms of contacts captured from a 3C or derivative experimental protocol. We assume Hi-C as the experimental protocol of choice, and the aim is to find $N$ 3D coordinates whose pairwise Euclidean distances correlate well with the contact frequency matrix $f \in{\mathbb{R}^{N \times N}}$. We can simplify the problem further by assuming contacts are binary [38], where the positive label could be thought of as a significant chromatin interaction, such as a detected chromatin loop [9].

**Figure 1:**
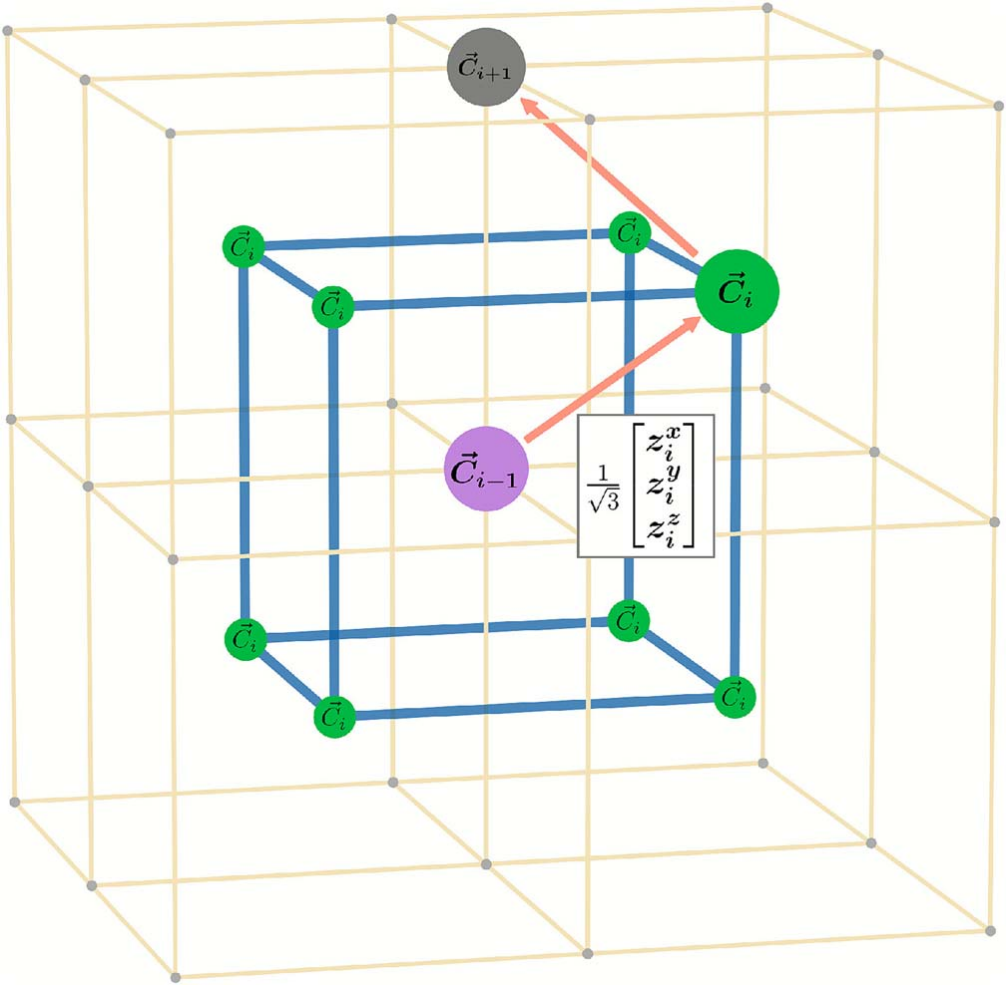
The eight possible locations for $C_{i}$ (green), with respect to the cube centered on $C_{i-1}$ (purple).

Genomic structures are threaded through a cubic lattice, with the first two beads fixed, where each bead $i$ can move to the eight possible corners of a cube centered at bead $i-1$. In other words, bead $i$’s location ($\vec{C}_{i}$) is a unit vector composed of three binary (spin) variables $z_{i}^{x},z_{i}^{y},z_{i}^{z} \in \{-1,1\}$, corresponding to the eight corners of a cube, translated by $\vec{C}_{i-1}$, see Fig. 1. The recursive and closed form definitions for the location of bead $i$, with $i> 1$, are defined by Equations (1) and (2), respectively.

Notice that $z_{i}^{x}$, $z_{i}^{y}$, and $z_{i}^{z}$ are not 3D coordinates, but binary variables representing these eight corners (three bits can represent in total $2^{3}=8$ states). For example, the biggest green bead in Fig. 1 can be associated with the binary (spin) string of (+1,+1,+1). In total, each structure is mapped to the state of $3(N-2)$ qubits. The reason for being $3(N-2)$ is because each bead requires three binary (spin) variables, and the first two beads’ locations are fixed. Therefore, each structure is represented as a string of binary (spin) variables of length $3(N-2)$. When we measure the quantum state $\ket{\psi }$ of these qubits, each qubit will result in either $-1$ or 1 (Z basis), which can be considered as 0 and 1. Thus, the entire measured state of all $3(N-2)$ qubits corresponds to a 3D structure. 


(1) \begin{align*} & \vec{C_{i}} = \vec{C}_{i-1} + \frac{1}{\sqrt{3}}\begin{bmatrix} z_{i}^{x} \\ z_{i}^{y} \\ z_{i}^{z} \end{bmatrix}\qquad\quad\ \ \end{align*}

(2) \begin{align*} & \vec{C_{i}} = \frac{1}{\sqrt{3}}\begin{bmatrix} 1 \\ 1 \\ 1 \end{bmatrix} + \frac{1}{\sqrt{3}} \sum_{k=2}^{i} \begin{bmatrix} z_{k}^{x} \\ z_{k}^{y} \\ z_{k}^{z} \end{bmatrix} \end{align*}

The squared Euclidean distance is defined as $D[i,j]^{2}$, see Equation (3). Note that by the properties of the lattice, $D[i,j]$ is bounded by $|i-j|$, i.e. $D[i,j]^{2} <= |i-j|^{2}$. 


(3) \begin{align*}& D[i,j]^{2} = ||\vec{C_{i}} - \vec{C_{j}}||_{2}^{2} = ||\vec{C_{i}}||_{2}^{2} + ||\vec{C_{j}}||_{2}^{2} - 2 * \vec{C_{i}} \cdot \vec{C_{j}}\end{align*}

From the Hi-C experiment, we can construct a contact matrix $\pi _{c}$. We assume a contact between beads $i$ and $j$ implies that the distance between the beads is less than or equal to some predefined radius $r$: 


(4) \begin{align*}& || \vec{C_{i}} - \vec{C_{j}} ||_{2} \leq r \iff \text{ Beads}\, i \text{ and}\, j \text{ are in contact.}\end{align*}

Let us define the entries of the contact matrix as $\pi _{c}[i,j]$, which gives the probability of a contact between beads $i$ and $j$. As previously mentioned, for simplicity we assume $\pi _{c}[i,j] \in \{0,1\}$, making $\pi _{c}$ a binary matrix. We construct the likelihood of $D[i,j]^{2}$ given $\pi _{c}[i,j]$ (Equation (5)) in terms of Equations (6) and (7), which correspond to attractive ($D[i,j]^{2}$ should approach $r^{2}$) and neutral propensities, respectively. 


(5) \begin{align*} & \text{P}\Big(D[i,j]^{2} \Big\lvert \pi_{c}[i,j]\Big) \propto f_{+}(i,j)^{\pi_{c}[i,j]}f_{-}(i,j)^{1-\pi_{c}[i,j]} \end{align*}

(6) \begin{align*} & f_{+}(i,j) = \Bigg(1 - \frac{|D[i,j]^{2} - r^{2}|}{|i-j|^{2} - r^{2}}\Bigg)^{\frac{|i-j|^{2}}{D[i,j]^{2}}}\qquad\quad \end{align*}

(7) \begin{align*} & f_{-}(i,j) = 1 - \frac{1}{1 + e^{4(D[i,j]^{2} - r^{2})}}\qquad\qquad\quad\ \end{align*}

**Figure 2:**
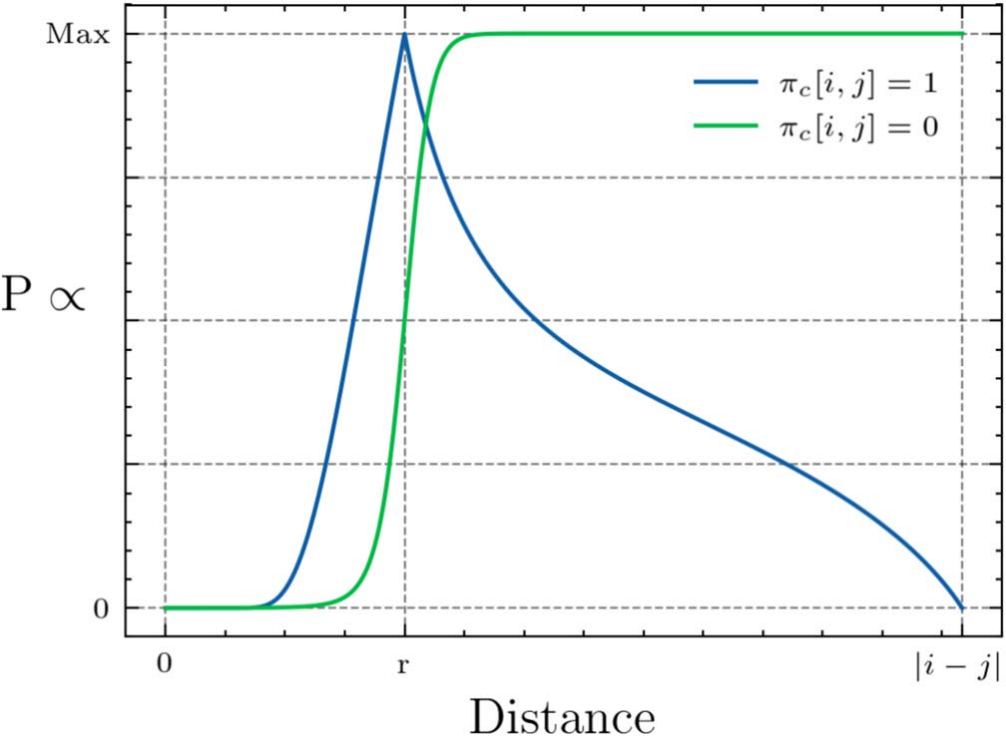
The likelihood of the distance ($D[i,j]$) between beads $i$ and $j$ given $\pi _{c}[i,j]$.

When a contact is not detected by the Hi-C experiment, i.e. $\pi _{c}[i,j] = 0$, the likelihood is an S-curve ($f_{-}$) that gives approximately equal plausibility for all distances except those that approach or are below the contact threshold $r$. When $\pi _{c}[i,j] = 1$, the likelihood is higher for distances near the contact threshold $r$ and lower otherwise ($f_{+}$), see Fig. 2.

For the $k$’th structure $\mathbb{S}_{k} \in \mathbb{R}^{N\times 3}$ in a group of structures $\mathbb{S}$, we model the likelihood of $\mathbb{S}_{k}$ in terms of its associated squared distance matrix $D^{2}_{k}$: 


(8) \begin{align*}& \text{P}\Big(D^{2}_{k} \Big| \pi_{c}\Big) \propto \prod_{i=0}^{N-1}\prod_{j=i+2}^{N-1}f_{+}(i,j)^{\pi_{c}[i,j]}f_{-}(i,j)^{1-\pi_{c}[i,j]}\end{align*}

Note that all of the $k$ structures are assumed to be generated from the quantum state $\ket{\psi (\theta )}$ parameterized by $\theta $. The angles $\theta $ are the parameters of the quantum gates that prepare the quantum state (a metaphor of $\theta $ are the parameters or weights of a neural network). More information regarding the parameterized quantum circuits can be found in Section “Choice of ansatz and optimizer”. The log-likelihood of Equation (8) is defined as the following: 


(9) \begin{align*}& \begin{split} \mathcal{L}(\theta) &=\log \text{P}\Big(D^{2}_{k} \Big| \pi_{c}\Big)\\ &=\sum_{i=0}^{N-1}\sum_{j=i+2}^{N-1}\left\{\begin{aligned} & (1-\pi_{c}[i,j])\log f_{-}(i,j) \\ & + (\pi_{c}[i,j])\log f_{+}(i,j) \\ & +\text{Const.} \\ \end{aligned}\right\} \end{split}\end{align*}

We further expand our model to account for the case of physical aggregations. Physical aggregations in the Hi-C experiment are the consensus non-single-cell contacts captured between genomic loci. Bulk Hi-C can be viewed as the average of single-cell Hi-C data [5]; thus, Equation (9) assumes zero aggregation. To incorporate aggregation into our model, we consider the case where multiple structures are sampled at a time (per-shot measurements from the VQA). Equations (10) and (11) define the criteria for aggregated and non-aggregated contacts over a collection of sampled structures, respectively. Note that $\underset{k}{\mathbb{E}}$ is the notation for the expected value (average value) of all the elements, with $k$ being the index of an element. 


(10) \begin{align*} & \underset{k}{\mathbb{\hat{E}}}[\mathcal{L}(\theta)] = \log \text{P}\Big(\underset{k}{\mathbb{E}}[D^{2}_{k}] \Big\lvert \pi_{c}\Big) \end{align*}

(11) \begin{align*} & \underset{k}{\mathbb{E}}[\mathcal{L}(\theta)] = \underset{k}{\mathbb{E}}\Big[\log \text{P}\big(D^{2}_{k} \big\lvert \pi_{c}\big)\Big] \end{align*}

The criteria of $\underset{k}{\mathbb{\hat{E}}}[\mathcal{L}(\theta )]$ (with a hat on top of $\mathbb{E}$), defined by Equation (10), computes the likelihood of the expected or averaged squared distances over a group of sampled structures. This would be used when trying to evaluate and or generate a population of structures that collectively match the input Hi-C data (population modeling). Optimizing over an objective function of the form $\underset{k}{\mathbb{\hat{E}}}[\mathcal{L}(\theta )]$ is highly applicable to VQAs, as such functions require access to multiple samples per optimization step, aligning well with the VQA procedure. The criterion $\underset{k}{\mathbb{E}}[\mathcal{L}(\theta )]$, defined by Equation (11), computes the likelihood of each squared distance matrix individually. This would be used with the goal of generating candidate structures that each individually best match the input Hi-C data.

In other words, for input bulk Hi-C data, when using Equation (10), we are generating a population of single-cell structures that, on average, match the input bulk Hi-C data. In contrast, when using Equation (11), we will generate consensus structures, each of which individually matches the input bulk Hi-C data. For further clarification, a flow chart describing the potential use cases for each criterion ($\alpha = 0$ and $\alpha = 1$) can be found in Supplementary Fig. S1. We define the generalized objective function over a group of sampled structures as: 


(12) \begin{align*}& \mathcal{L}(\theta, \alpha) = -(1 - \alpha) \underset{k}{\mathbb{E}}[\mathcal{L}(\theta)] - \alpha\underset{k}{\mathbb{\hat{E}}}[\mathcal{L}(\theta)]\end{align*}

The value of $\alpha \in [0,1]$ can be set depending on the level of aggregation one would like to model. Higher and lower values of $\alpha $ closer resemble the bulk and single-cell Hi-C experiments, respectively. When we increase the value of $\alpha $, we observe an increase in the diversity and variety of sampled structures; for more details, see Section “Results.” When $\alpha = 1$, we can interpret the sampled structures as an approximate subset of single-cell structures, from which the bulk Hi-C experiment was performed, i.e., a deconvolutional method. More details pertaining to the impact of $\alpha $ on the objective function landscape, learned distributions, and respective results can be found in Section “Results.”

Note that the power, $(\frac{|i-j|^{2}}{D[i,j]^{2}})$, in Equation (6) was intended for shorter-length structures ($N < 150$). As the genomic distance between two beads increases, the spatial clash penalization from the attractive propensity (Equation (6)) becomes more weak. One way to address this could involve converting Equation (6) into a piecewise function, or clipping the values of $D[i,j]$ and $|i-j|$ in the exponent term. The current quantum simulators and devices also create a limitation of $N \leq 12$. Classical computers cannot support high qubit counts due to memory limitations. Furthermore, we found that $\gtrsim 32$ qubits will generate too much noise on a physical device (regardless of the number of available qubits), making the system unstable.

### Choice of ansatz and optimizer

**Figure 3:**
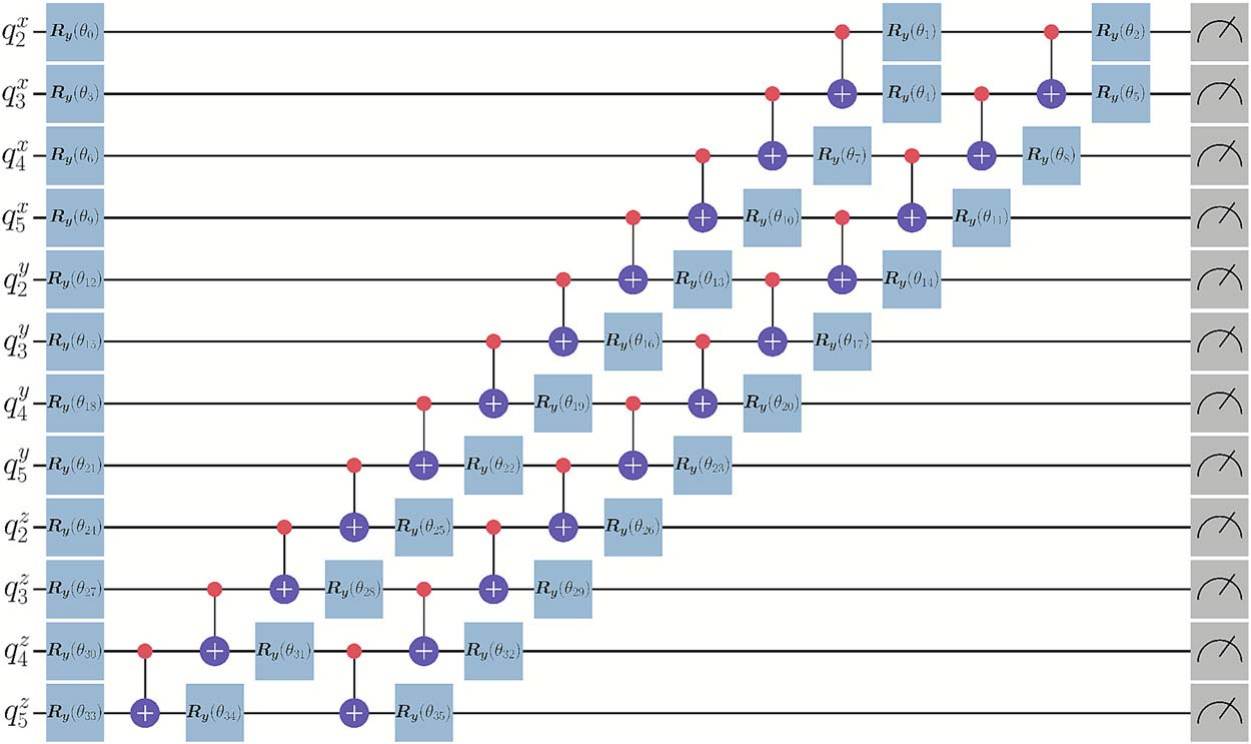
Two repetitions of the RealAmplitudes ansatz with reverse linear entanglement for a 12-qubit system with length $N=6$ structures. Each red dot (control) and the plus symbol below it (target) represents the control NOT (CNOT) gate. Each blue box represents a $R_{y}$ quantum gate (y-axis rotation gate). The gray box at the end of each wire corresponds to a measurement (Z-basis). The whole quantum circuit diagram is read from left to right (applying gates simultaneously column-wise).

The Hamiltonian corresponding to $\underset{k}{\mathbb{E}}[\mathcal{L}(\theta )]$ can be described entirely by Pauli Z ($\sigma _{z}$) operators [92, 93]. Similarly, the Hamiltonian corresponding to $\underset{k}{\mathbb{E}}[D^{2}_{k}]$ is an Ising model, and is also composed entirely of $\sigma _{z}$ operators. From these observations, we can restrict our quantum state space to contain strictly real amplitudes. For simplicity, we utilized two repetitions of the RealAmplitudes ansatz provided by Qiskit [94]. In Fig. 3, we display the quantum circuit diagram when modeling a length 6 structure (12 qubits). The first two beads’ locations are fixed, so these 12 qubits represent the locations of four DNA beads. For example, $q^{x}_{2}$ represents a qubit, which corresponds to the binary variable used to define the location of the third DNA bead in the X direction relative to the second DNA bead. Each qubit has three parameters associated with it, e.g. $q^{x}_{2}$ is associated with $\theta _{0}$, $\theta _{1}$, and $\theta _{2}$. The number of parameters, $p$, to model $N$ beads on a string is $p = 9(N - 2)$. For the choice of optimizer, we used Constrained Optimization by Linear Approximation (COBYLA) [95] since the values of $p$ experimented with were relatively small. Parameters were initialized randomly over the uniform distribution with range $[0, 2\pi )$.

As more capable quantum machines come to fruition, it will be necessary to more thoroughly investigate both the choice of classical optimization algorithm, ansatz, and parameter initialization strategies. Exploring these areas is outside the scope of this research. For more information on these topics and their implications with regard to VQAs, we refer to Refs. [79, 96–102].

### Variational quantum algorithm for generating genomic conformations

**Figure 4:**
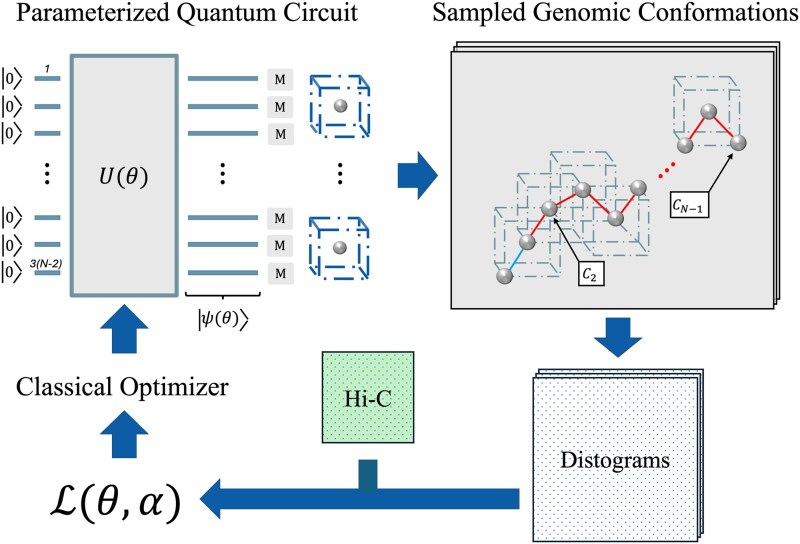
VQA for generating genomic conformations. The $U(\theta )$ is the ansatz, representing the parameterized quantum circuit shown in Fig. 3. The $\ket{0}$ indicates the default input state of each qubit.

We now state the proposed VQA for the 3D genome reconstruction problem. First, we randomly initialize $\theta \in [0,2\pi )$ and prepare the quantum state $\ket{\psi (\theta )}$ using the parameterized ansatz. Then, we sample $\rho $ measurements, $\mathbb{M}$, from $\ket{\psi (\theta )}$. We map the measurements $\mathbb{M}$ to structures $\mathbb{S}^{\prime}$ and compute the cost $\mathcal{L}(\theta , \alpha )$ over $\mathbb{S}^{\prime}$. The cost and current $\theta $ are passed to a classical optimization algorithm to update $\theta $. The process repeats until convergence. We can generate an ensemble of viable genomic conformations by preparing and sampling from $\ket{\psi (\theta ^{*})}$. See Fig. 4 and Algorithm 1.

Note that all of the Pauli strings needed to compute $\mathcal{L}(\theta , \alpha )$ mutually commute with one another, thereby allowing for simultaneous measurements [103, 104]. It takes $\mathcal{O}(N^{2}\rho )$ time, with $\rho $ being the number of measurements (samples), to compute $\mathcal{L}(\theta , \alpha )$.



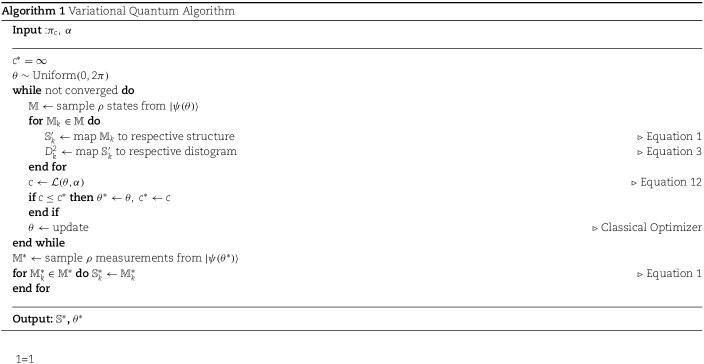



## Results

### Simulated genomic contact dataset

To evaluate whether our proposed algorithm can effectively learn the conformational space, we generated multiple simulated Hi-C datasets. Let us define the set of length $N$ structures as $\mathbb{S}$, with $\mathbb{S}_{k} \in \mathbb{R}^{N \times 3}$ and $|\mathbb{S}| = 2^{3(N-2)} = 8^{N-2}$. We were able to generate, via brute force, all possible structures with $N \in [6,11)$ that are free from spatial clashes (all pair-wise distances are $\geq \frac{1}{4}$). We partitioned each $\mathbb{S}$ into a variety of subsets (groups) $\mathcal{G} \subset \mathbb{S}$. Topological properties are extracted from each $\mathcal{G}$ and used to define the ground truth for the objective function $\mathcal{L}(\theta ,\alpha )$.

Specifically, we defined subsets of structures through common pair-wise contacts. Two beads $i$ and $j$ are in contact when their Euclidean distance is within a predefined radius $r$, see Equation (4). For our experiments, we set $r = 1.5$. Groups are formally defined as follows: 


(13) \begin{align*} & \xi(\mathbb{S}_{k}) = \left\{(i,j) \text{} \Bigg\lvert \text{} \begin{aligned} &||\vec{C}_{i} - \vec{C}_{j}||_{2} \leq r\\ &|i-j|> r\\ \end{aligned}\text{} \right\}\qquad \end{align*}

(14) \begin{align*} & \mathcal{G}_{\chi} = \left\{\mathbb{S}_{k} \in \mathbb{S} \text{} \Bigg\lvert \text{} \begin{aligned} &\underset{i,j}{\text{all}}\text{:} ||\vec{C}_{i} - \vec{C}_{j}||_{2} \geq \frac{1}{4} \\ &\chi=\xi(\mathbb{S}_{k})\\ \end{aligned}\text{} \right\} \end{align*}

The contacts related to each group can be viewed as the result of a Hi-C experiment, where a contact implies a loop between genomic loci $i$ and $j$. For a group $\mathcal{G}_{\chi }$, the respective ground truth is defined as the following: 


(15) \begin{align*}& \pi_{c}({i,j}) = \begin{cases} 1 & \text{If}\, (i,j) \in \chi \\ 0 & \text{Else} \end{cases}\end{align*}

#### Evaluation from simulation of quantum circuits on a classical computer

In our experiments, we set the number of shots $\rho = 4096$. We then randomly sampled targets $\mathcal{G}_{T} \subset \mathbb{S}$ for structures of lengths $N \in [6,11)$. We ran our VQA 10 times per target over as many targets that were computationally feasible. To verify the performance of our algorithm, and the subsequent impact of the parameter $\alpha $, we computed multiple evaluation metrics over sampled ensembles of $4096$ structures, $\mathbb{S}_{k} \in \mathbb{S}^{*}$, from $\ket{\psi (\theta ^{*})}$.

To directly evaluate the ability of our algorithm for finding likely conformations, for each target group, we computed all of the possible theoretical likelihoods (Equation (8)) across the $8^{N-2}$ states, each of which is associated with an energy level. Higher likelihoods are associated with lower energy levels and vice versa. We define the ground truth distribution vector, $\phi (\pi _{c})$, as follows: 


(16) \begin{align*}& \phi(\pi_{c}) = [P(D^{2}_{0} | \pi_{c}),\cdots, P(D^{2}\, {8^{N-2}} | \pi_{c})]\end{align*}

From $\phi (\pi _{c})$, we computed the structures in the upper $q$ quantile of the likelihoods. We then compared those against the $4096$ sampled structures $\mathbb{S}_{k} \in \mathbb{S}^{*}$. Coverage is defined as the percent of the total states in the upper $q$’th quantile, $\mathbb{S}[\phi (\pi _{c}) \geq Q(\phi ,q)]$, sampled by the algorithm: 


(17) \begin{align*}& \text{Coverage} = \frac{|\mathbb{S}[\phi(\pi_{c}) \geq Q(\phi,q)]\text{} \cap\text{} \mathbb{S}^{*}|}{|\mathbb{S}[\phi(\pi_{c}) \geq Q(\phi,q)]|}\end{align*}

The empirical probability given $q$ is the observed chance for a sampled structure $\mathbb{S}_{k} \in \mathbb{S}^{*}$ to be at or above the upper quantile $q$. In Supplementary Fig. S3, we plot coverage and the empirical probability for quantiles in the range of $[0.5, 1)$ for structures of length $N = 10$.

It is clear from Supplementary Fig. S3B that the empirical likelihood of sampling a structure in the top-50th percentile (quantile) is $\sim 100\%$. However, this chance decreases as the quantile increases for all values of $\alpha $, with the rate of decrease being more significant for larger values of $\alpha $. Supplementary Fig. S3A and B indicate that higher values of $\alpha $ can hinder the ability to sample structures with the exact target contacts, but can improve the ability to sample a variety of structures with reasonable similarity to the target contacts (Bulk Hi-C).

**Figure 5:**
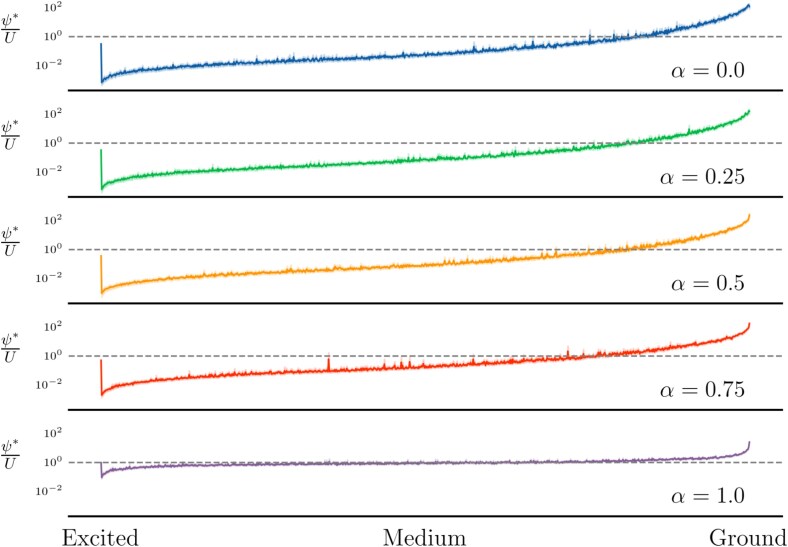
Binned line plot for the likelihood ratio of the learned quantum distribution ($\psi ^{*}$) over the uniform distribution (U), partitioned by excitation (energy) level, for length $N=10$ structures. The shadow behind the lines indicates a 95% confidence interval.

In Fig. 5, we plot the likelihood ratio ($\frac{\psi ^{*}}{U}$) of the learned quantum distribution ($\psi ^{*}$) over the uniform distribution ($U$) partitioned by excitation level—equivalent to the likelihood (Equation (8)). When $\frac{\psi ^{*}}{U}> 1$, the learned probability ($\psi ^{*}$) of sampling a structure with a given excitation level is $\frac{\psi ^{*}}{U}$ times more likely compared to random chance ($U$).

What we observe is that smaller values of $\alpha $ tend to more drastically reward and penalize lower- and higher-energy solutions, respectively. Large values of $\alpha $ still penalize high-energy solutions, but are more lenient towards medium to lower-energy solutions. In other words, higher values of $\alpha $ give leeway to solutions that are not perfect, but are still reasonably likely given the contact data. This suggests that by increasing, $\alpha $ we focus the algorithm on sampling a more diverse, evenly distributed set of states across the medium-to-low-energy spectrum (bulk Hi-C). Hence, as we decrease, $\alpha $ we notice the sampled structures are more highly concentrated and focused around the lowest energy states of the system (single-cell Hi-C). It is worth noting that, in general, a large proportion of states are high-energy, see Supplementary Fig. S7.

To further support these findings, we plot the Shannon–Entropy ($H$) [105] curves of our VQA for structures of length $N = 6$, see Supplementary Fig. S2. What we notice is that as more optimization steps (lower costs) are performed, the decrease in Shannon–Entropy of the learned probability distribution is much larger for smaller values of $\alpha $. The distribution with maximal entropy is the uniform distribution, which suggests larger values of $\alpha $ associate with more evenly distributed learned quantum states.

We expand upon these results by evaluating inferred contact probabilities from the sampled pairwise distances. Structures that contain any pairwise distance $ < \frac{1}{4}$ (spatial clash) are ignored. The inferred contact probability given the pairwise distance $D[i,j]$ was motivated by Schuette *et al.* [27] and is defined as the following: 


(18) \begin{align*}& \tilde{\pi}_{c}[i,j] = \begin{cases} 1 & \text{If}\ D[i,j] \leq r \\ \left(\frac{r}{D[i,j]}\right)^{3} & \text{Else} \end{cases}\end{align*}

We evaluate the inferred contact probabilities $\tilde{\pi }_{c}(i,j)$ by calculating the average precision (AP) [106] and area under the curve (AUC) [106]. We set the average inferred contact probabilities of the positive- and negative-labeled contacts as the positive and negative predictions, respectively, thereby making the labels perfectly balanced. We also computed the maximum and mean of the average inferred probabilities for positively labeled contacts. All four metrics are displayed in Supplementary Fig. S6 for targets with lengths $N \in [6,11)$.

What we observe supports the relation of $\alpha $ to the single-cell and bulk Hi-C experiments. When $\alpha $ is smaller (single-cell), more precise inferred contacts are recovered. When modeling a bulk Hi-C experiment, more variations not only in the conformations are expected (single-cell), but also in the recovered contacts, as the contacts are captured from a large heterogeneous set of single-cell conformations [107] (Bulk Hi-C). In other words, we expect and observe the precision and ranking metrics (Supplementary Fig. S6A and B) to be stronger for lower values of $\alpha $ (single-cell), and the recall metrics (Supplementary Fig. S6C and D) to be stronger for higher values of $\alpha $ (Bulk Hi-C).

We also define two variants of the Dice-Sørensen Index (DSI) [108, 109], weighted (DSI$_{w}$) and maximum-threshold (DSI$_{m}$), see Equations (19) and (20). Both assess the contact–recovery performance, with higher values indicating a stronger ability to recover target contacts. 


(19) \begin{align*} & {\text{DSI}}_{w} = \frac{2{\sum}\pi_{c}(i,j)\tilde{\pi}_{c}(i,j)}{{\sum}\pi_{c}(i,j) + \tilde{\pi}_{c}(i,j)}\qquad\qquad\qquad\qquad\qquad \end{align*}

(20) \begin{align*} & {\text{DSI}}_{m} = \text{Max}\left\{ \frac{2{\sum}\pi_{c}(i,j)\text{I}(\tilde{\pi}_{c}(i,j)> t)}{{\sum} \pi_{c}(i,j) + \text{I}(\tilde{\pi}_{c}(i,j) > t)}\text{} \Bigg\lvert\text{} \forall t \in \tilde{\pi}_{c} \right\}\ \end{align*}

From Supplementary Fig. S4, it is clear that the aggregation parameter $\alpha $ can cause changes in the performance metrics, where both $\alpha =0$ and $\alpha =1$ are not optimal. This suggests that the ability to model and fine-tune the amount of aggregation can offer notable advantages, depending on the use case.

#### Landscape analysis of the aggregation parameter $\alpha $

To gain further insights into the effect of modeling aggregation, we performed exploratory landscape analysis and principal component analysis (PCA) [110] over $\mathcal{L}(\theta , \alpha )$. Our findings suggest $\mathcal{L}(\theta , \alpha )$ becomes less rugged as we increase $\alpha $.

**Figure 6:**
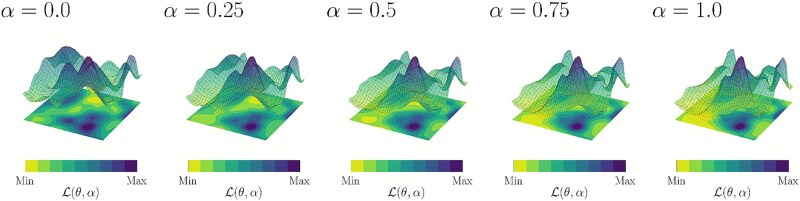
PCA embeddings of the learned parameter landscapes with $\alpha = 0$ for a single target group of length $N=6$ structures. The PCA embeddings are fixed, with the Z-axis denoting the loss function $\mathcal{L}(\theta ,\alpha )$. The loss function for each landscape is calculated explicitly with different values of $\alpha $ to show the change in local minima of $\mathcal{L}(\theta ,0)$ as $\alpha $ is increased. Each loss is independently normalized to the same scale (linearly), allowing for clear comparison.

Following the procedure described by Pérez-Salinas *et al*. [88], we computed the empirical maximum information content ($H_{M}$) over the parameter space. The number of parameters, $p$, was used to determine the number of samples, $128p$. Similarly, we computed $64$ random walks each with a length of $64p$. We notice that $H_{M}$ tends to decrease as we increase $\alpha $, see Supplementary Fig. S6. The value of $H_{M}$ is related to the ruggedness of the landscape [88, 111, 112], with higher values suggesting a more rugged landscape, and hence are generally more trainable. We further observe this visually through PCA embeddings of the objective function landscape (see Fig. 6).

As we increase the value of $\alpha $, the landscape becomes flatter and evenly distributed around the local minima. Generally, this would be viewed as an increase in optimization landscape difficulty. However, we observed in Section “Evaluation from simulation of quantum circuits on a classical computer” that the proposed algorithm still behaves as expected for different values of $\alpha $. This suggests that, depending on the use case and problem instance, the gained utility of increasing $\alpha $ could outweigh the slight increase in the difficulty of landscape optimization.

### Evaluation on experimentally obtained genomic contact data

To further validate our methodology, we modeled the 3D structure of a low-resolution bulk Hi-C matrix with the proposed VQA. Captured contact frequency and Euclidean distance form an inverse power law relationship [28–30]: 


(21) \begin{align*}& D[i,j] \propto f[i,j]^{-\lambda}\end{align*}

The optimal power $\lambda> 0$ can vary between datasets. Let us denote the minimum non-zero contact frequency detected between loci $i$ and $i+1$ as $f_{\text{unit}}$. We define the contact matrix $\pi _{c}$ in the following way: 


(22) \begin{align*}& \pi_{c}[i,j] = \begin{cases} 1 & \text{If}\, \frac{f[i,j]}{f_{\text{unit}}} \geq r^{(-\frac{1}{\lambda})} \\ 0 & \text{Else} \end{cases}\end{align*}

Similar to Equation (18), we define the inferred contact probability, $\tilde{\pi }_{c}[i,j]$, given the pairwise distance $D[i,j]$ as follows: 


(23) \begin{align*}& \tilde{\pi}_{c}[i,j] = \begin{cases} 1 & \text{If}\ D[i,j] \leq r \\ \left(\frac{r}{D[i,j]}\right)^{\left(\frac{1}{\lambda}\right)} & \text{Else} \end{cases}\end{align*}

We used Cooler [113] to produce a low-resolution (10 MB) balanced contact matrix of chromosome 18 for the GM12878 cell-line [9], see Table 1.

**Table 1:** Genomic data configurations for the GM12878 cell-line

Chromosome	Resolution (MB)	Polymer length	$\lambda $	No. contacts
18	10	8	$1/3$	17
18	10	8	$1/2$	11
18	10	8	$2/3$	8

For every configuration defined in Table 1, we ran 10 trials of our algorithm with noise-induced simulators and a noise-free simulator, as in Section “Evaluation from simulation of quantum circuits on a classical computer.” We then prepared and sampled the optimal pretrained parameters for each of the 10 trials to see if the classically learned parameters could effectively replicate the distribution of learned genomic conformations on a real quantum device. We ran our experiments on the physical ${ibm{\_}sherbrooke}$ [94] quantum device, of which the noise was modeled during simulations. However, due to high computational resource utilization for noisy-simulation and long wait times for access to the real quantum device, we only performed experiments with $\alpha =0$ and $\alpha =1$.

We first investigate the empirical Shannon Entropy ($\tilde{H}$) for the set of sampled states $\mathbb{S}^{*}$, see Equation (24) and Supplementary Fig. S8. We observe the same patterns in Supplementary Fig. S8 as in Supplementary Fig. S2. However, it is clear that the empirical entropy from the noisy simulator and real device is higher than that of the ideal parameters on the noise-free simulator. This is expected, as noise naturally increases the uncertainty of the distribution and hence increases the entropy. This suggests that, in general, noise impacts the learned distribution and thus more precise devices can improve the utility of the samples. 


(24) \begin{align*}& \tilde{H} = - \sum_{s \in \mathbb{S}^{*}} \text{Pr}(s)\log_{2}(\text{Pr}(s))\end{align*}

**Figure 7:**
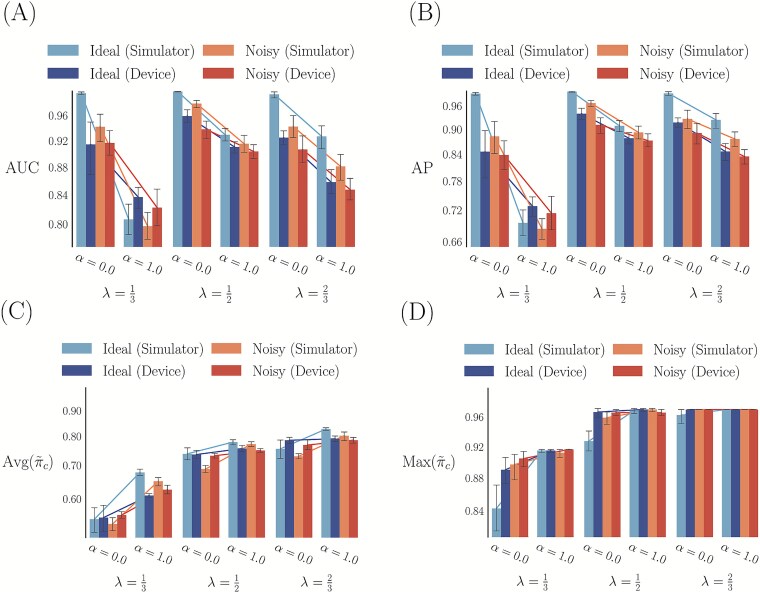
Bar plot with 95% confidence interval of the AUC (A), AP (B), average (C), and maximum (D) inferred contact probabilities. Parameters ($\theta $) were found through Qiskit simulations [94], with the optimal parameters ($\theta ^{*}$) run on both the simulators and the ${ibm{\_}sherbrooke}$ [94] quantum device.

In Fig. 7, we plot the AUC, AP, and recall metrics for the samples from the optimal parameters on the real device and learned parameters on the simulators (ideal and noisy). The metrics were computed as in Supplementary Fig. S6. Similar to the empirical entropy analysis, we also observe the same patterns in Fig. 7 as in Supplementary Fig. S6. Interestingly, while noise can affect the entropy of the distribution significantly, it has less of an effect on the expected trends for AUC, AP, and recall measures. This demonstrates that even in the presence of noise, the learned parameters can still resemble the distribution of conformations for single-cell ($\alpha = 0$) and bulk ($\alpha = 1$) Hi-C experiments. While it is still necessary to conduct larger-scale experiments in the future, given access to and improvements in quantum hardware, these are promising and encouraging results.

For the samples from $\alpha = 0$, we evaluated them using the Spearman rank correlation [114] and scale-invariant root mean squared deviation (RMSD) to the target distances. We computed the RMSD as Supplementary Equation S2. To assess the performance against classical consensus reconstruction methods, we compared the best sampled consensus structures against the classical consensus method Flamingo [37]. We used the precomputed 5 kb resolution structure provided by Flamingo [37] and downsampled the 3D model (average pooling) to 10 MB resolution.

**Figure 8:**
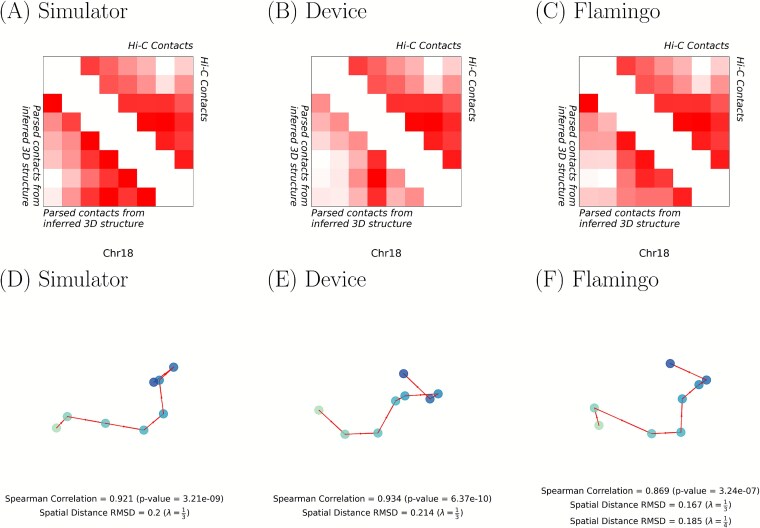
Visualization of results when modeling the consensus 10 MB chromosome 18 structure of GM12878 $\left (\lambda = \frac{1}{3}\right )$ using our algorithm on the ideal classical simulator, the “ibm_sherbrooke” quantum device, and Flamingo [37]. (A–C) Triangular heatmap displaying the contacts parsed from the inferred 3D structure, the true Hi-C contacts, and (D–E) the inferred consensus 3D structure.

Figure 8 displays, with $\lambda = \frac{1}{3}$, the best consensus inferred 3D structure based on both the Spearman correlation and spatial distance RMSD. The respective spatial distance RMSD and Spearman correlation between the contacts parsed from the inferred 3D structure and those obtained from the Hi-C data are stated below each structure. Both our method and Flamingo [37] find consensus structures that fit the target contact frequency matrix, as indicated by the spatial distance RMSD values. Furthermore, the Spearman correlations for our methods and Flamingo [37] are strong with values $>0.85$ and respective $P$-values $<10^{-6}$.

To further evaluate the heterogeneity of the sampled conformations when performing population modeling tasks ($\alpha = 1$), we compared our sampled conformations against real-world single-cell Hi-C data [115]. We calculated the paired F1 contact recovery scores [106] and the Jensen–Shannon (JS) divergence between the two distributions. Further details on how we approximated and compared the distributions can be found in the Supplementary, Section S.1. We then calculated the same metrics using the precomputed population structures from IGM [32]. HiCLift [116] was used to convert the single-cell matrices from hg19 to hg38 when evaluating with the precomputed structures from IGM [32].

**Figure 9:**
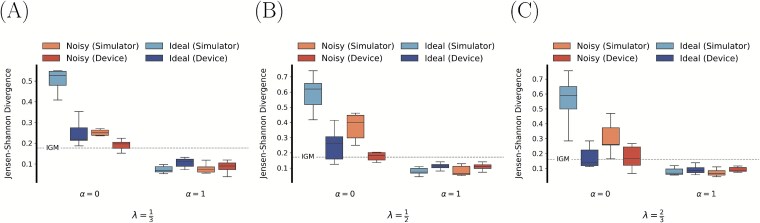
Estimated JS divergence between kernel density estimations (KDE) of the UMAP [117] (hamming distance) embeddings for binary contact matrices from the true single-cell conformations and sampled single-cell conformations. Sampled contact matrices were calculated using Equation (4). True contact matrices were determined using Equation (22) with corresponding values of (A) $\lambda = \frac{1}{3}$, (B) $\lambda = \frac{1}{2}$, and (C) $\lambda = \frac{2}{3}$.

Figure 9 displays box plots of the JS divergence between the true and inferred single-cell distributions across all values of $\alpha $ and $\lambda $. Distributions were approximated using KDE on UMAP [117] (hamming distance) embeddings of binary contact matrices. When performing population modeling ($\alpha $ = 1), we see for all four cases tight inter-quartile ranges with median JS divergences $<0.2$, indicating general agreement between the inferred and true population of contact matrices. Note that the respective observed median divergences, for each case, are higher when performing consensus modeling ($\alpha = 0.0$). Furthermore, the population modeling results are in alignment with those of IGM [32], where the JS divergences are also below $0.2$, i.e., $0.177$, $0.173$, and $0.161$, for $\lambda = \frac{1}{3}, \frac{1}{2}, \frac{2}{3}$, respectively.

We also computed paired F1 contact scores (linear sum assignment problem) using a variant of the Jonker–Volgenant algorithm [118, 119]. What we observe further solidifies our algorithm’s ability to replicate the distribution of single-cell conformations, with hundreds of uniquely paired samples (excluding duplicates) achieving F1 scores above 0.7. Supplementary Fig. S9 displays for all four cases the average paired sample percentage above the F1 score threshold (x-axis). Furthermore, when, $\alpha = 0$ the sampled conformations do not consistently approximate the distribution of single-cell conformations, and the number of paired counts decreases, highlighting the efficacy of the aggregation parameter $\alpha $.

Using the same procedure, we also applied our algorithm when modeling a topologically associating domain (TAD) boundary. We ran our algorithm with $\alpha = 0.0$ on a sub-matrix centered on a TAD-boundary at 25 kb resolution. Our target matrix comprised of 10 bins located within chromosome 11 of GM12878. The system required 24 qubits to model the 10 bead structure. Due to the high-qubit count of this problem, we simulated the system using a noiseless simulator of quantum circuits on a classical device. The more stable “ibm_torino” quantum device was used to sample conformations using the learned optimal parameters.

Supplementary Fig. S10 displays, from both the simulator and physical quantum device, the parsed contact matrix and insulation scores from the inferred 3D structure and the epigenetic signal of CTCF binding sites captured by ChIP-seq [120, 121]. We then determined the best sampled consensus structure in terms of the combination of spatial distance RMSD and Spearman correlation. The best structure, for both the simulator and physical device, achieved a strong Spearman rank correlation $>0.9$ with $P$-values $<10^{-6}$. Furthermore, with TAD boundaries being related to CTCF binding sites [122], it is clear that the insulation scores parsed from both best sampled 3D structures are inversely related to the CTCF epigenetic signal, supporting the sampled structures' validity. These results reinforce the ability of our algorithm in recovering likely consensus structures that capture the structural and epigenetic properties of a localized TAD boundary.

### Evaluation including inter-chromosomal genomic contact data

For further analysis of the future applicability of our algorithm, we extended our algorithm to model both intra- and inter-chromosomal interactions within the nucleus. Due to computational resource limitations, we modeled a two-polymer system. Each individual polymer was grown from both sides of a fixed reference coordinate. We then discretized the distance between the two reference points to allow for conformational variability. The complete mathematical details of the modifications can be found in Supplementary Section S2.

We modeled the consensus structure ($\alpha $ = 0) of chromosomes 5 and 8 using Yeast Hi-C data [123] at 1 MB resolution. This corresponds to a 12-bead system (two six-bead polymers) that, with the aforementioned algorithmic modifications, requires 32 qubits. Due to computational resource limitations, we ran three trials of our algorithm on a noiseless classical simulator of quantum circuits. We further investigated the real-world performance by running the learned optimal parameters on a real quantum device. Due to the high-qubit count of this problem, we used the more stable “ibm_torino” quantum device. When using the classical simulator, the intra-chromosomal inferred and target contact matrices showed strong Spearman correlations of $0.8$ and $0.9$, with $P$-values $<10^{-2}$, for chromosomes 5 and 8, respectively. The parsed inter-chromosomal contact matrix from the inferred 3D structure showed a moderate Spearman correlation of $0.69$ with a $P$-value of $3.168 \times 10^{-6}$. All three correlations degraded by $\sim 0.1$ when run on the physical quantum device, which is expected due to system noise. The results are summarized in Supplementary Fig. S11.

## Discussion and conclusion

In this paper, we propose a novel VQA for reconstructing 3D genome structures from experimental genomic contact data. Our extensive analysis supports the notion that our algorithm can effectively model and sample from the distribution of genomic conformations. We have also shown that our methodology can theoretically model the conformational space expected for both single-cell and bulk experiments.

One inherent limiting factor when developing VQAs is the current lack of high-qubit and high-fidelity physical quantum devices. In this paper, we used the COBYLA [95] optimizer due to the small size of the problem instances that we experimented with. However, as the number of parameters increases, it will be necessary to use a more memory efficient and noise resilient optimization algorithms, such as Simultaneous Perturbation Stochastic Approximation (SPSA) [124] or gradient-based algorithms, such as Limited-memory Broyden–Fletcher–Goldfarb–Shanno (L-BFGS) [125]. Note that the gradient with respect to the quantum circuit parameters can be computed using the parameter-shift rule [126, 127]. Again, due to the small size of our problem instances, we utilized two repetitions of the RealAmplitudes [94] ansatz. While this ansatz is reasonable in the current context of this study, some further avenues of exploration, given sufficient hardware, could involve using deeper repetitions or Hamiltonian (dependent on the problem instance) inspired designs [98]. Still, the performance of these methods remains unclear without further hardware innovations.

There are several potential benefits for reconstructing 3D genome structures with a VQA, particularly when modeling conformational ensembles. When training the proposed VQA, the number of parameters grows linearly with respect to the number of genomic loci, which is only slightly larger than that needed to store a few genomic conformations classically. Thus, when modeling bulk Hi-C data, we can deconvolve genomic structures into an ensemble without having the optimization memory requirements (parameters) grow with respect to the size of the ensemble, i.e., the number of parameters, $9(N-2)$, depends only on the number of beads ($N$) per structure. To model a population of M structures with N beads on a string in 3D space, classical population modeling algorithms, such as IGM [32], generally require at least $3MN$ parameters, which can be particularly computationally demanding in terms of both memory and compute, especially when modeling large populations ($M$) in high-resolution ($N$). This same idea applies to single-cell Hi-C data, where we embed a likely ensemble of genomic structures corresponding to a single-cell Hi-C matrix. Furthermore, sampling from a quantum machine is in principle very efficient and could offer benefits compared with parallel classical sampling, e.g., simulated annealing.

The results from this study serve as a starting point for highlighting the conceivable impact quantum computation can have on 3D genomics. We expect future advancements in quantum technology to come with much promise for insights into the 3D genome and its respective regulatory landscape.

**Key Points**

- Variational quantum algorithms (VQAs) can effectively model the distribution of 3D genome conformations.
- The proposed methodology exhibits expected trends for single-cell and population-based experiments.
- The insights from this study provide the foundation for modeling 3D genome conformations with VQAs.

## Supplementary Material

supplementary_bbaf663

## Data Availability

The datasets generated for Fig. 7 and Supplementary Figs S6, S4, and S8 are available in the GitHub repository https://github.com/zwang-bioinformatics/3D-genome-VQA. All other datasets are available from the corresponding author on request.
