# Supplementary material for: Generating three-dimensional genome structures with a variational quantum algorithm
Source: Brief Bioinform. 2025 Dec 15;26(6):bbaf663. doi: 10.1093/bib/bbaf663 (PMC12704447; doi:10.1093/bib/bbaf663)
Supplement: supplementary_bbaf663 [file supplementary_bbaf663.pdf]

Supplementary document

Generating three-dimensional genome structures with a variational quantum algorithm

Andrew Jordan Siciliano[1] and Zheng Wang[1,*]

[1]Department of Computer Science, University of Miami, 1365 Memorial Drive, Coral Gables, 33124, FL, USA
[*]Correspondence: zheng.wang@miami.edu

$$scale(f, D, \lambda) = \frac{\sum_{i=0}^{N-1}\sum_{j=i+2}^{N-1} D[i,j]\left(\frac{f[i,j]}{f_{\text{unit}}}\right)^{-\lambda}}{\sum_{i=0}^{N-1}\sum_{j=i+2}^{N-1} D[i,j]^2} \tag{S.1}$$

$$\text{RMSD}(f, D, \lambda) = \sqrt{\left(\frac{2\sum_{i=0}^{N-1}\sum_{j=i+2}^{N-1}\left(scale(f,D,\lambda)D[i,j] - \left(\frac{f[i,j]}{f_{\text{unit}}}\right)^{-\lambda}\right)^2}{(N-1)(N-2)}\right)} \tag{S.2}$$

S.1 Single-cell conformational distribution approximations

We used two methods to approximate and compare the experimental and inferred distributions of single-cell conformations. Both methods involve approximations, as we only have access to samples from the experimental and inferred distributions.

To approximate and compare the inferred and true single-cell contact distributions, we projected each binary contact matrix to a low-dimensional (2D) embedding using UMAP [1] with the Hamming distance metric. Our low-level embeddings were fit to the space of all possible contact matrices formed from valid structures (no spatial clashes). We then used Gaussian Kernel Density Estimation (KDE) to approximate the experimental and inferred distributions of the conformations. We then evaluated the density of points within a grid of 64x64 points. We treated these density values as the relative likelihood of a point. We then divided each density by the sum of all densities to construct an approximate probability vector. These are the vectors used when computing the Jensen-Shannon divergence.

To generate the optimal pairings between inferred and true single-cell contact matrices, we used a variant of the Jonker-Volgenant algorithm [2, 3]. The distance between two samples is the Hamming distance. Once the optimal pairings were determined, we computed the F1 scores for each pair. We then calculated the percentage of unique contact matrices above each F1-score threshold.

S.2 Extension of algorithm for two polymers

S.2.1 Location reformulation

As in the single-polymer case, each bead's location is defined in terms of three binary (spin) variables. However, we fix one bead from each of the two polymers P_0 and P_1, instead of two beads. We denote these two indices as the junction site J, where each bead $\vec{C}_i^p \in P_p$ is grown from the ends of the respective junction bead, $\vec{C}_{J[p]}^p$, for the polymer p.

$$\vec{C}_i^{\,p} = \frac{1}{\sqrt{3}}\begin{bmatrix} z_i^x \\ z_i^y \\ z_i^z \end{bmatrix} + \begin{cases} \vec{C}_{i-1}^p & i > J[p] \\ \vec{C}_{i+1}^p & i < J[p] \end{cases} \tag{S.3}$$

To position the two polymers, we discretize the relative distance between junction sites using the base-10 representation of a k bit state $\mathcal{U}_2$.

$$\mathcal{D} = \sqrt{3} + \frac{\mathcal{U}_2}{\sqrt{3}} - \frac{1}{4} \tag{S.4}$$

We define the fixed positions of the junction sites as the following:

$$\vec{C}^0_{J[0]} = \begin{bmatrix} 0 \\ 0 \\ 0 \end{bmatrix} \tag{S.5}$$

$$\vec{C}^1_{J[1]} = \begin{bmatrix} \mathcal{D} \\ 0 \\ 0 \end{bmatrix} \tag{S.6}$$

Note that with these definitions, the minimum possible Euclidean distance between any two non-homologous beads is $\frac{1}{4}$, which helps to limit spatial clashes. This two-polymer system is represented with $3(|P_1|+|P_0|-2)+k$ qubits. In our experiments, we set $k = 2$ and chose the junction sites as the indices of the maximum inter-chromosomal contact frequency.

S.2.2 Likelihood function reformulation

Based on the above definitions, we extend our likelihood function to account for both inter- and intra-chromosomal contacts. Inter-chromosomal contacts are defined using the minimum f_{unit} of the two polymers. Notice that the distance between any two beads with indices (i, j) in this system is bounded by the following:

$$\mathcal{B}[i,j] = \begin{cases} \left\| \left(\vec{C}^0_{J[0]} - \frac{|i-J[0]|}{\sqrt{3}} \begin{bmatrix} 1 \\ 1 \\ 1 \end{bmatrix} \right) - \left(\vec{C}^1_{J[1]} + \frac{|j-J[1]|}{\sqrt{3}} \begin{bmatrix} 1 \\ 1 \\ 1 \end{bmatrix} \right) \right\|_2 & (i \in P_0 \;\; \& \;\; j \in P_1) \\ \left\| \left(\vec{C}^0_{J[0]} - \frac{|j-J[0]|}{\sqrt{3}} \begin{bmatrix} 1 \\ 1 \\ 1 \end{bmatrix} \right) - \left(\vec{C}^1_{J[1]} + \frac{|i-J[1]|}{\sqrt{3}} \begin{bmatrix} 1 \\ 1 \\ 1 \end{bmatrix} \right) \right\|_2 & (i \in P_1 \;\; \& \;\; j \in P_0) \\ |i-j| & \textit{else} \end{cases} \tag{S.7}$$

Since both inter and intra-contacts contribute to the likelihood of the system, we redefine the radius r as the following:

$$r(i,j) = \begin{cases} \sqrt{3} & (i \in P_0 \;\; \& \;\; j \in P_1) \vee (i \in P_1 \;\; \& \;\; j \in P_0) \\ 1.5 & \textit{else} \end{cases} \tag{S.8}$$

We then extend the neutral and attractive propensity definitions to the following:

$$f_+(i,j) = \left(1 - \frac{|D[i,j]^2 - r(i,j)^2|}{\mathcal{B}[i,j]^2 - r(i,j)^2} \right)^{\frac{\mathcal{B}[i,j]^2}{D[i,j]^2}} \tag{S.9}$$

$$f_-(i,j) = 1 - \frac{1}{1 + e^{4(D[i,j]^2 - r(i,j)^2)}} \tag{S.10}$$

To account for the different cardinality of inter- and intra-chromosomal pairs, we weight the likelihood function with respect to the total pairs L.

$$L = \left(|P_0 \times P_1| + \frac{1}{2} \sum_{m \in [0,1]} (|P_m| - 1)(|P_m| - 2) \right) \tag{S.11}$$

The weights ($\gamma(i,j)$) for indices i, j are defined as the following:

$$\gamma(i,j) = \begin{cases} \frac{L}{|P_0 \times P_1|} & (i \in P_0 \;\; \& \;\; j \in P_1) \;\; \& \;\; (i < j) \\ \\ \frac{2L}{(|P_0|-1)(|P_0|-2)} & (i, j \in P_0) \;\; \& \;\; (i < j+1) \\ \\ \frac{2L}{(|P_1|-1)(|P_1|-2)} & (i, j \in P_1) \;\; \& \;\; (i < j+1) \\ \\ 0 & else \end{cases} \tag{S.12}$$

We then define the new weighted likelihood function as the following:

$$\mathrm{P}\Big(P_0, P_1 \Big| \pi_c\Big) \propto \prod_{i,j} \Big(f_+(i,j)^{\pi_c[i,j]} f_-(i,j)^{1-\pi_c[i,j]}\Big)^{\gamma(i,j)} \tag{S.13}$$

S.3 Figures

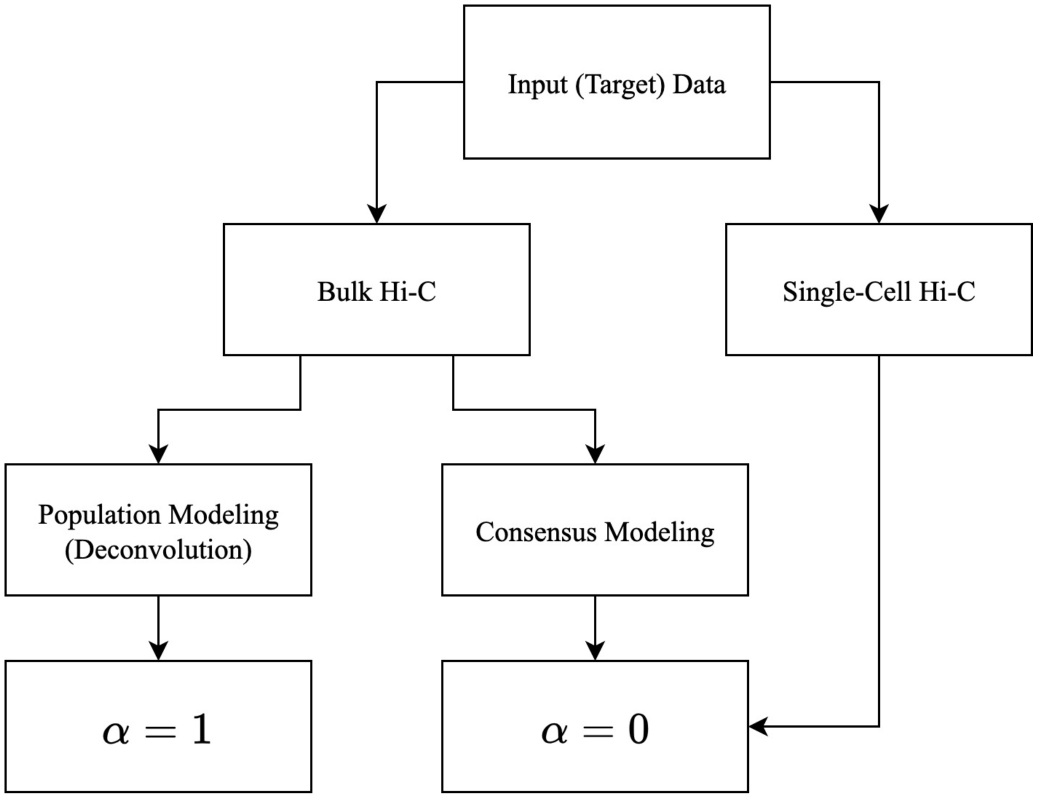

Figure S.1: Flow chart depicting potential use cases for each criterion ($\alpha = 0$ and $\alpha = 1$).

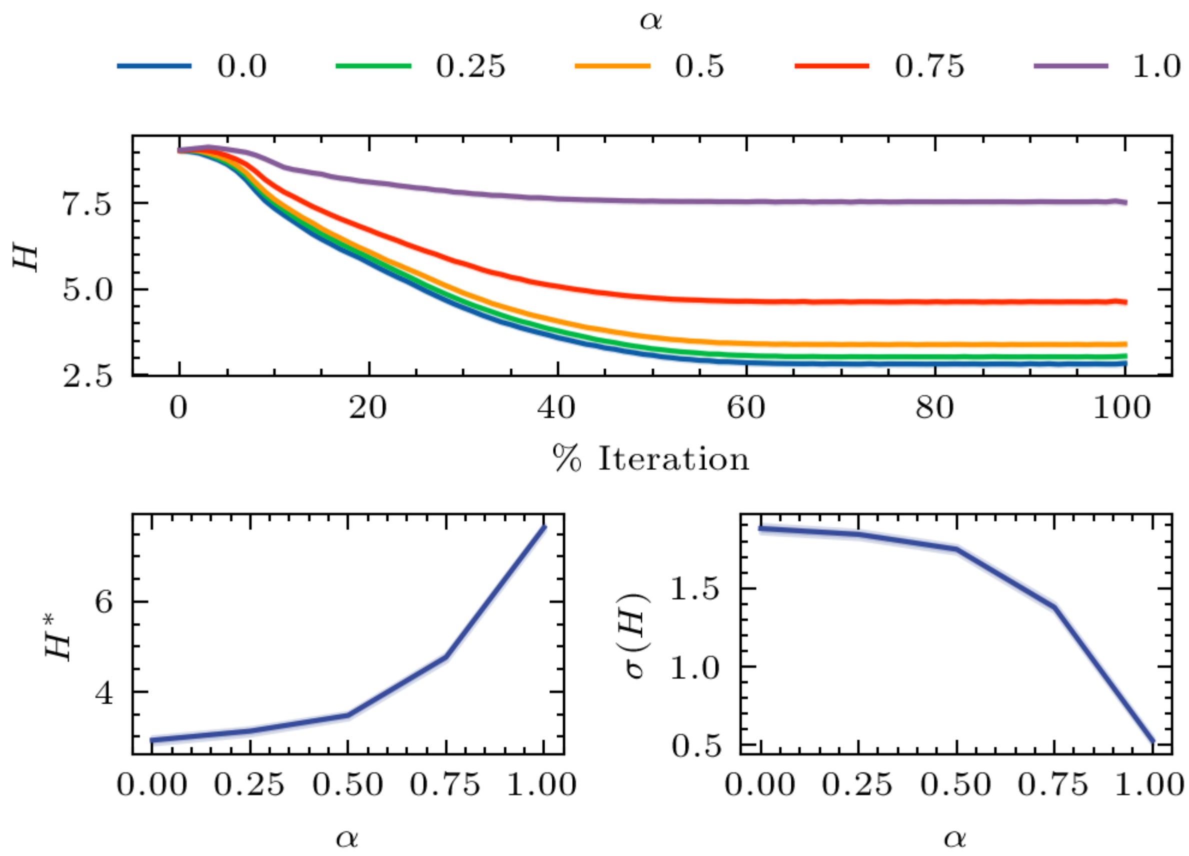

Figure S.2: Line plots for the Shannon Entropy (H) per % iteration, converged H (H^*), and standard deviation of H ($\sigma(H)$) during training for structures of length $N = 6$. The shadow behind the lines indicates a 95 percent confidence interval.

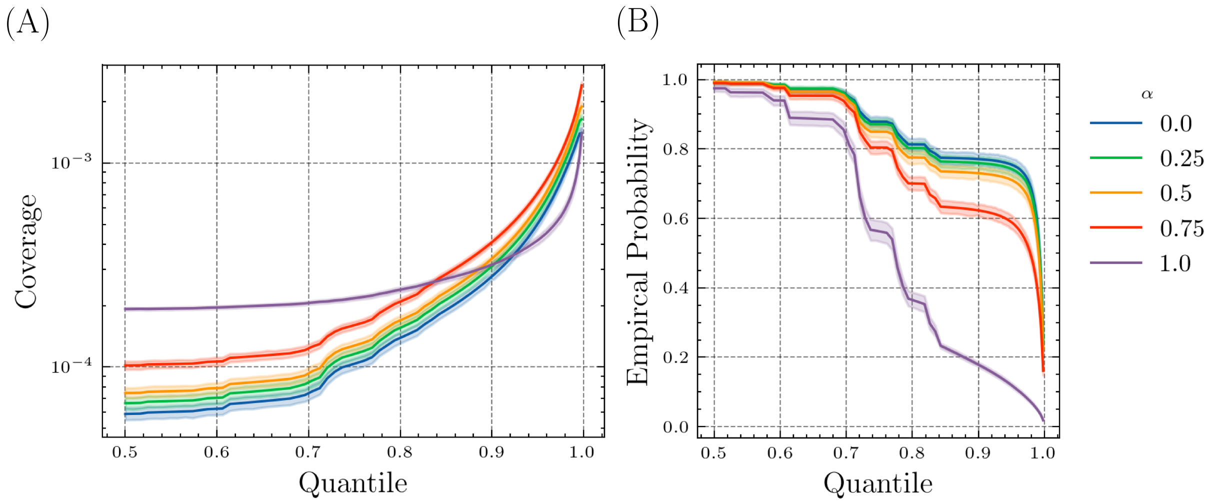

Figure S.3: Line plot and 95 percent confidence interval of the Coverage (A) and Empirical Probability (B) given q (quantile) for structures of length 10 over 48 target groups.

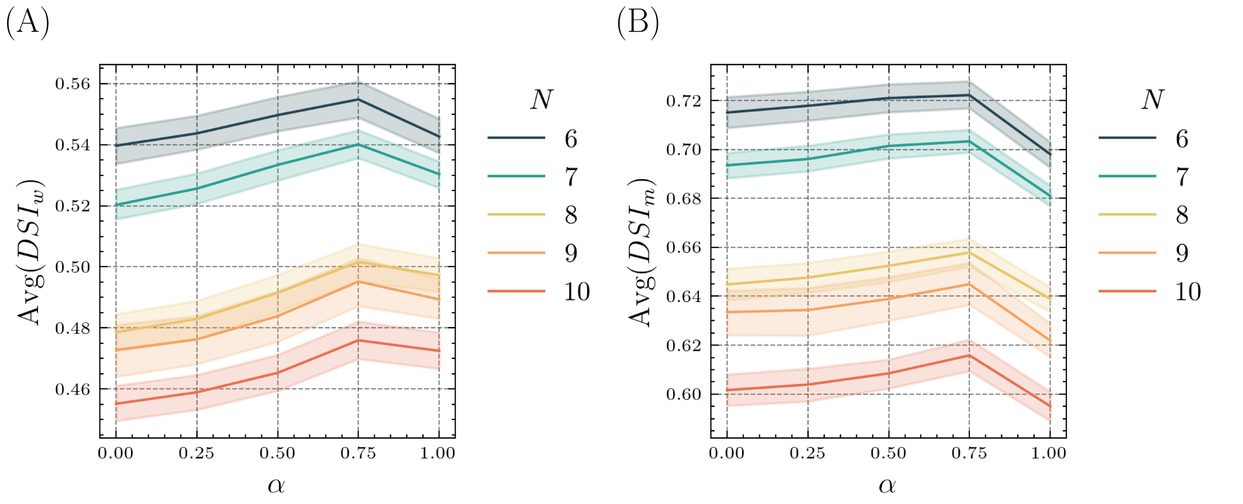

Figure S.4: Line plot and 95 percent confidence interval of the weighted (A) and maximum threshold (B) Dice-Sørensen index [4, 5].

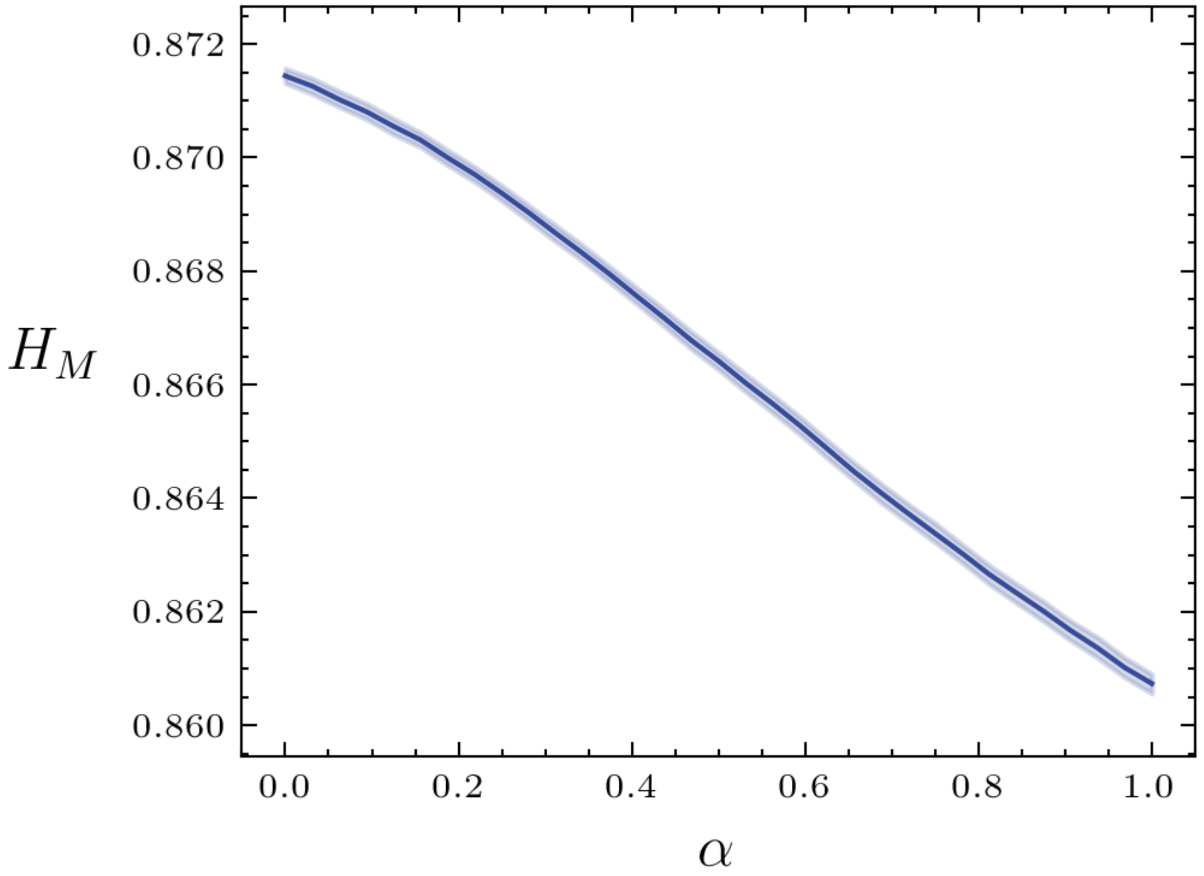

Figure S.5: Line plot and 95 percent confidence interval of empirical H_M values across a random sample of groups of length 6 structures.

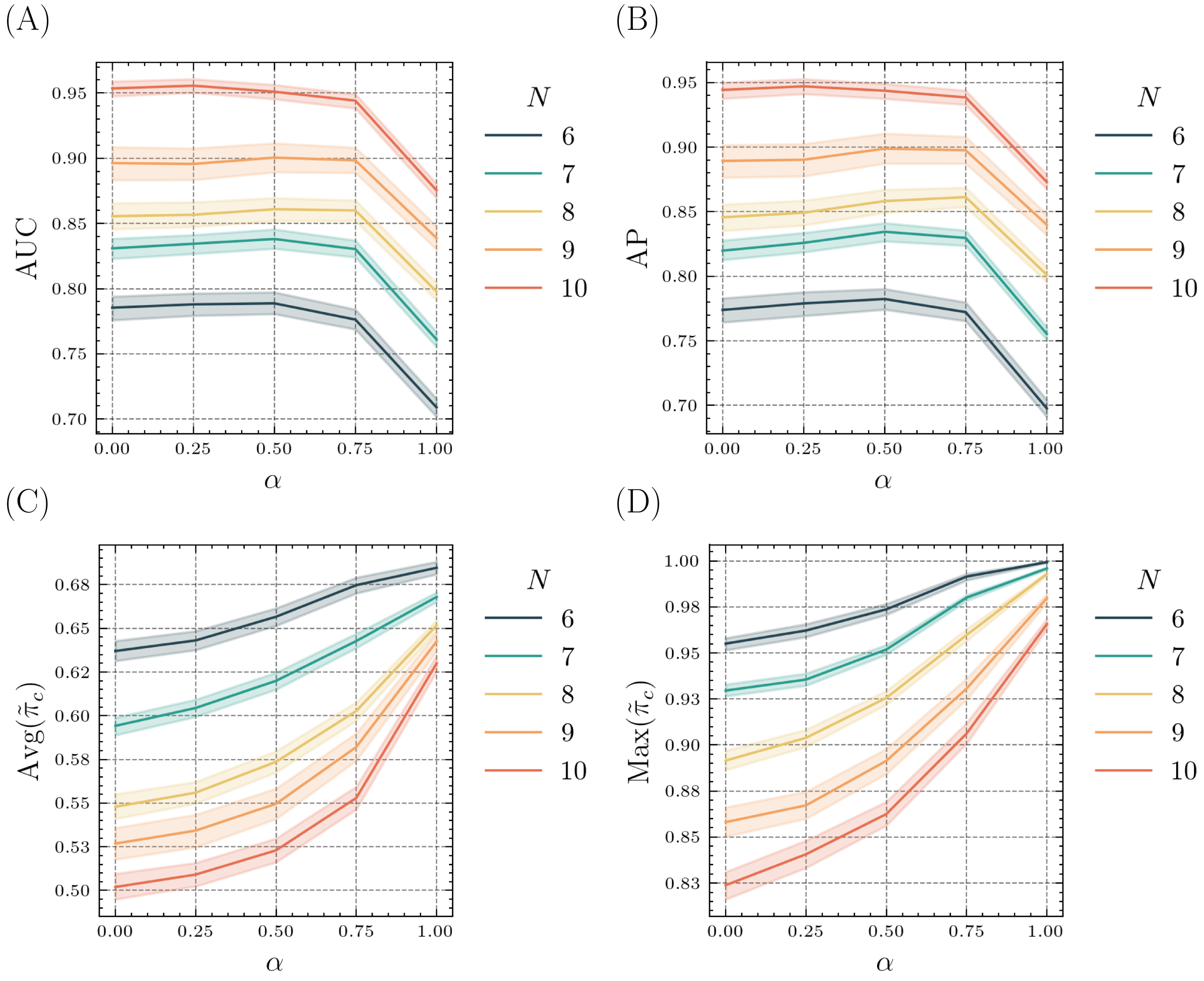

Figure S.6: Line plot and 95 percent confidence interval of the area under the curve (A), average precision (B), average (C), and maximum (D) inferred contact probabilities.

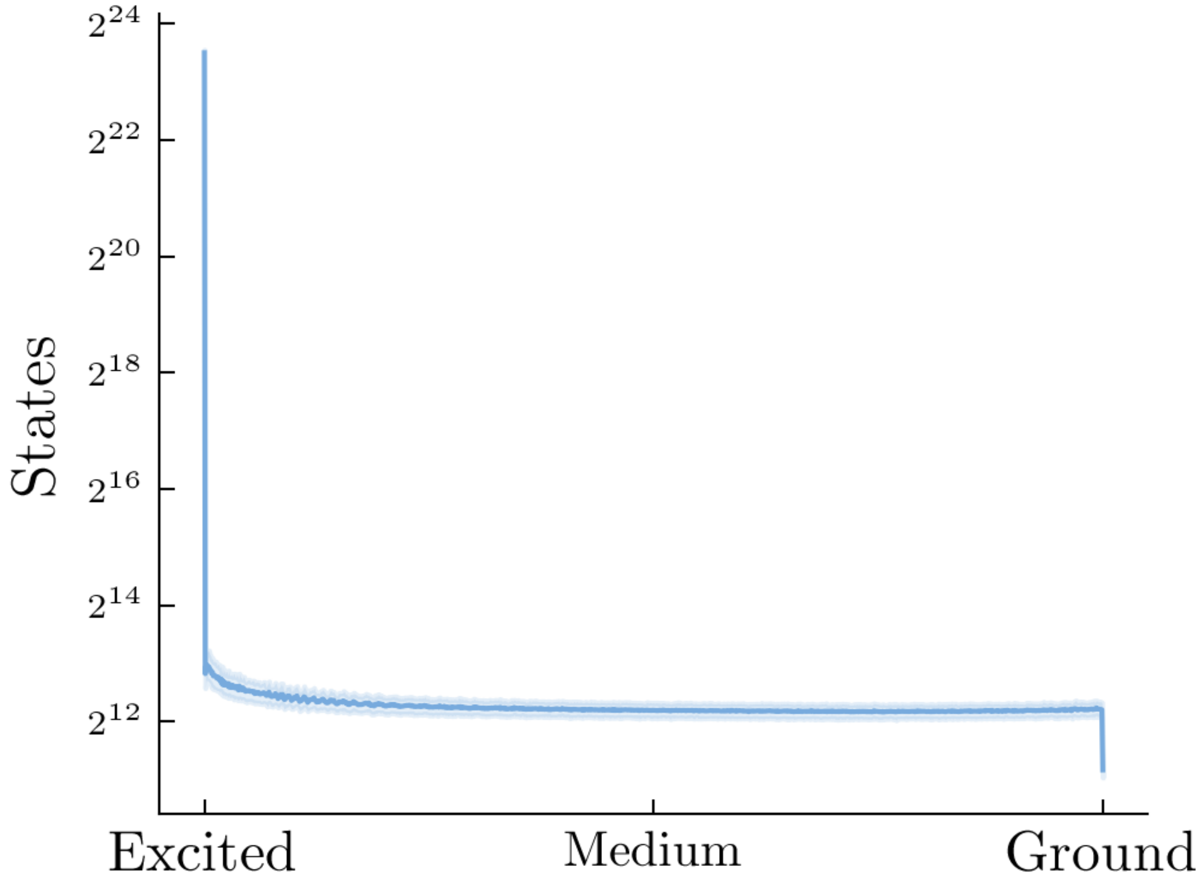

Figure S.7: Line plot and 95 percent confidence interval of the energy distribution of states, partitioned by excitation level, over 48 target groups with length $N = 10$ structures.

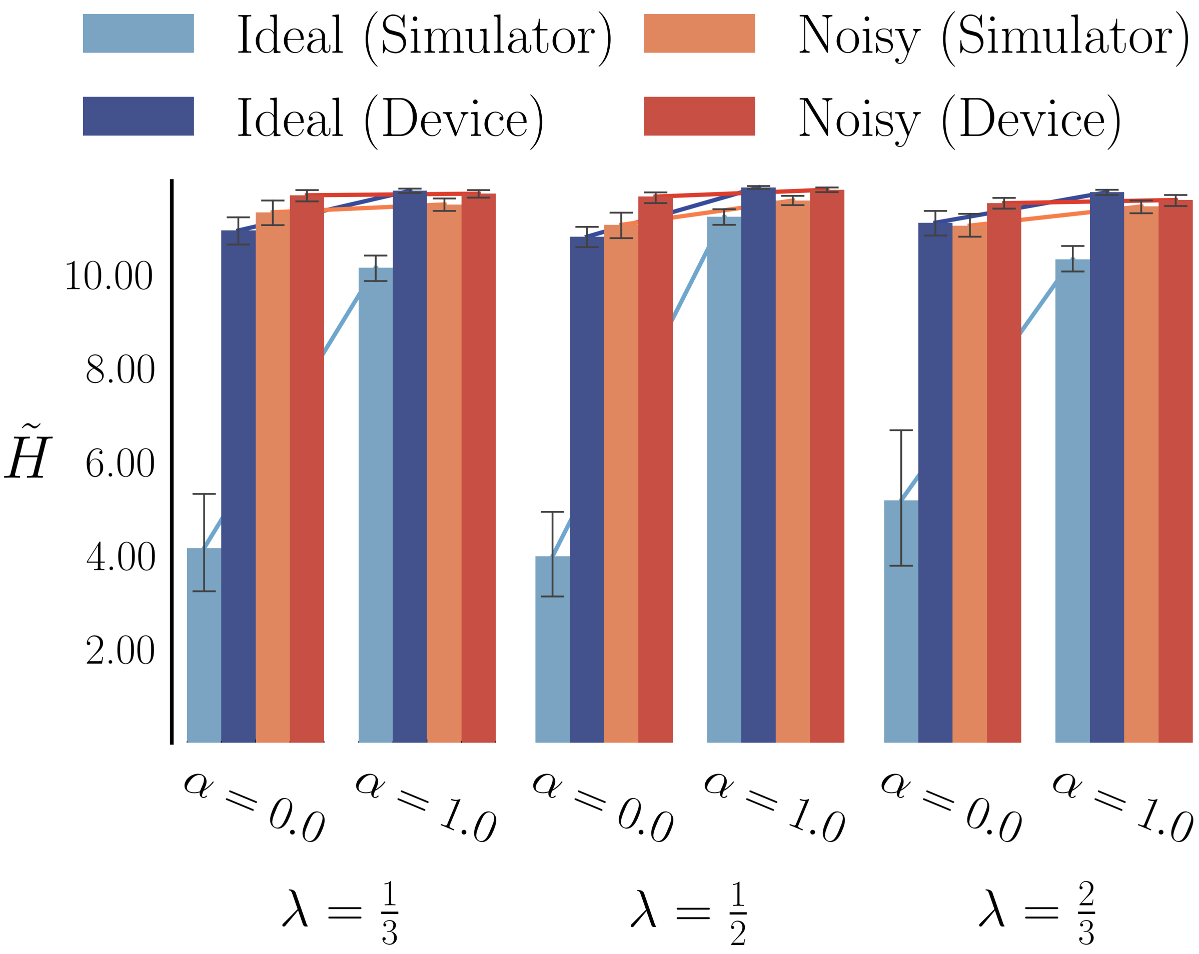

Figure S.8: Bar plot with 95 percent confidence interval of the empirical entropy ($\tilde{H}$).

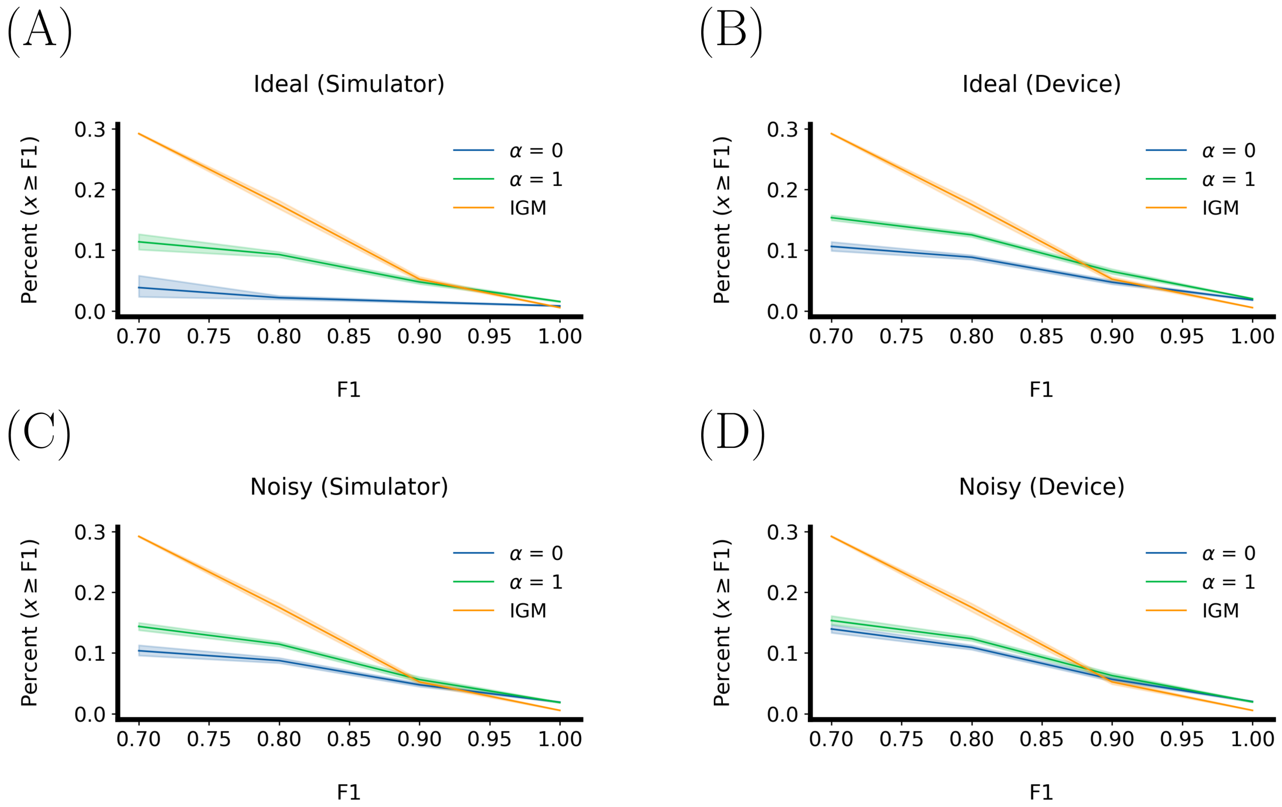

Figure S.9: Percent of F1 thresholded uniquely paired samples (by linear hamming distance sum assignment [2, 3]) from the (A) ideal simulator, (B) ideal device, (C) noisy simulator, and (D) noisy device. Each percentage (y-axis) corresponds to the percent of uniquely paired contact matrices (percent is with respect to the size of the inferred population) greater than or equal to each F1 threshold (x-axis). Note that IGM generated a population of 10000 structures and our algorithm generated a population of 4096 potential structures (ignoring structures with spatial clashes). The shadow behind the lines indicates a 95 percent confidence interval.

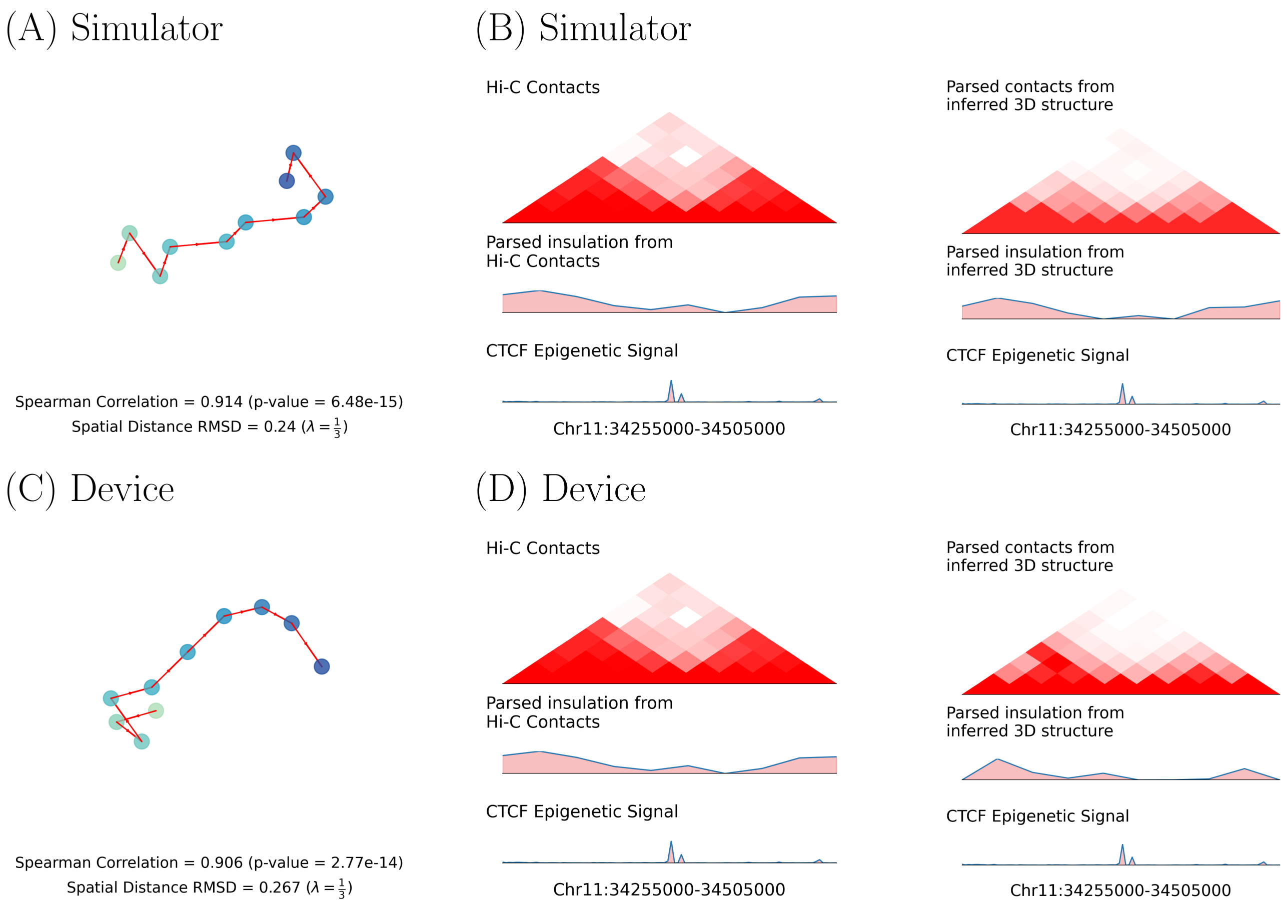

Figure S.10: Visualization of results when modeling a 25kb TAD boundary located within chromosome 11 of GM12878 with $\lambda = \frac{1}{3}$. (A) inferred 3D structural model from the best sampled consensus structure when performing classical simulation of quantum circuits. (B) Triangular matrix plot of the contacts from the Hi-C data and parsed from the inferred 3D structure, insulation scores from the Hi-C data and parsed from the inferred 3D structure, and CTCF epigenetic signal when performing classical simulation of quantum circuits. (C) The same as (A) but on when using the physical quantum device "ibm_torino" prepared with the learned optimal parameters. (D) The same as (B) but on when using the physical quantum device "ibm_torino" prepared with the learned optimal parameters.

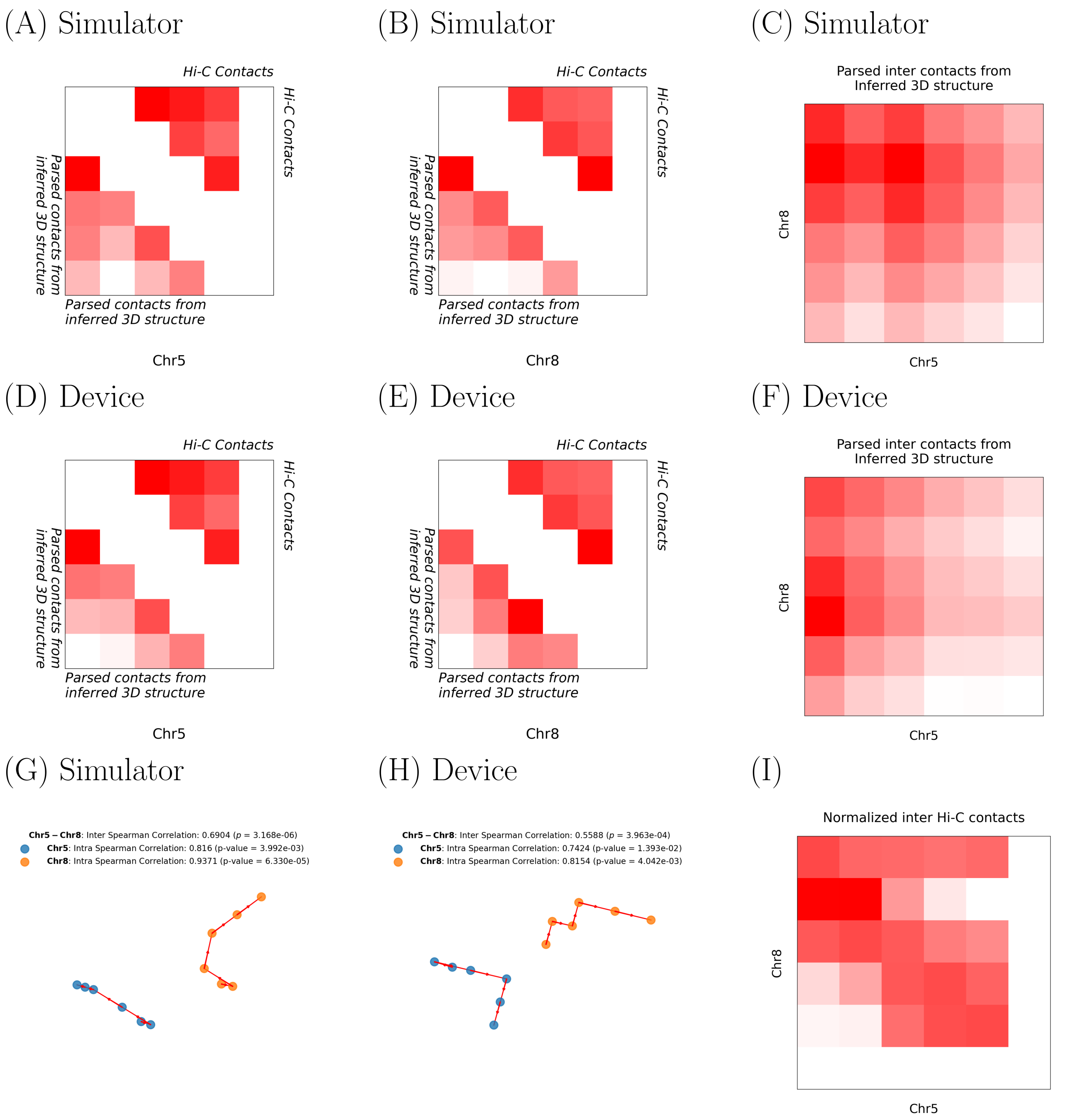

Figure S.11: Results from the simulator and device when modeling a two-polymer system of Yeast Hi-C data with $\alpha = 0.0$. Learned optimal parameters from the simulator were ran on the "ibm_torino" quantum device. Inferred (parsed from the 3D structure) and true chromosomal contacts for intra-chromosomal 5 (A, D) and intra-chromosomal 8 (B, E). Inter-chromosomal contacts between chromosomes 5 and 8 parsed from the inferred 3D structure (C, F) and from the Hi-C data (I). Best sampled 3D structure, based upon Spearman correlations, from the simulator (G) and quantum device (H).

References

[1] Leland McInnes, John Healy, and James Melville. Umap: Uniform manifold approximation and projection for dimension reduction. *arXiv preprint arXiv:1802.03426*, 2018.

[2] David F Crouse. On implementing 2d rectangular assignment algorithms. *IEEE Transactions on Aerospace and Electronic Systems*, 52(4):1679–1696, 2016.

[3] Pauli Virtanen, Ralf Gommers, Travis E Oliphant, Matt Haberland, Tyler Reddy, David Cournapeau, Evgeni Burovski, Pearu Peterson, Warren Weckesser, Jonathan Bright, et al. Scipy 1.0: fundamental algorithms for scientific computing in python. *Nature methods*, 17(3):261–272, 2020.

[4] Lee R Dice. Measures of the amount of ecologic association between species. *Ecology*, 26(3):297–302, 1945.

[5] Thorvald Julius Sørensen. *A method of establishing groups of equal amplitude in plant sociology based on similarity of species content and its application to analyses of the vegetation on Danish commons.* I kommission hos E. Munksgaard, 1948.
